# The weak land carbon sink hypothesis

**DOI:** 10.1126/sciadv.adr5489

**Published:** 2025-09-10

**Authors:** James T. Randerson, Yue Li, Weiwei Fu, Francois Primeau, Jinhyuk E. Kim, Mingquan Mu, Forrest M. Hoffman, Anna T. Trugman, Linqing Yang, Chao Wu, Jonathan A. Wang, William R. L. Anderegg, Alessandro Baccini, Mark A. Friedl, Sassan S. Saatchi, A. Scott Denning, Michael L. Goulden

**Affiliations:** ^1^ Department of Earth System Science, University of California, Irvine, CA 92697, USA.; ^2^ Department of Civil and Environmental Engineering, University of California, Irvine, CA 92697, USA.; ^3^ Department of Geography, University of California, Los Angeles, CA 90095, USA.; ^4^ Computational Sciences and Engineering Division and Climate Change Science Institute (CCSI), Oak Ridge National Laboratory, Oak Ridge, TN 37831, USA.; ^5^ Department of Geography, University of California, Santa Barbara, CA 93106, USA.; ^6^ Wilkes Center for Climate Science and Policy, University of Utah, Salt Lake City, UT 84112, USA.; ^7^ School of Biological Sciences, University of Utah, Salt Lake City, UT 84112, USA.; ^8^ Chloris Geospatial, 399 Boylston Street, Boston, MA 02116, USA.; ^9^ Department of Earth and Environment, Boston University, Boston, MA 02215, USA.; ^10^ CTrees, 12 S Raymond Ave., Pasadena, CA 91105, USA.; ^11^ Jet Propulsion Laboratory, California Institute of Technology, Pasadena, CA 91109, USA.; ^12^ Department of Atmospheric Science, Colorado State University, Fort Collins, CO 80523, USA.

## Abstract

Over the past three decades, assessments of the contemporary global carbon budget consistently report a strong net land carbon sink. Here, we review evidence supporting this paradigm and quantify the differences in global and Northern Hemisphere estimates of the net land sink derived from atmospheric inversion and satellite-derived vegetation biomass time series. Our analysis, combined with additional synthesis, supports a hypothesis that the net land sink is substantially weaker than commonly reported. At a global scale, our estimate of the net land carbon sink is 0.8 ± 0.7 petagrams of carbon per year from 2000 through 2019, nearly a factor of two lower than the Global Carbon Project estimate. With concurrent adjustments to ocean (+8%) and fossil fuel (−6%) fluxes, we develop a budget that partially reconciles key constraints provided by vegetation carbon, the north-south CO_2_ gradient, and O_2_ trends. We further outline potential modifications to models to improve agreement with a weaker land sink and describe several approaches for testing the hypothesis.

## Teaser

Satellite-derived time series of vegetation carbon do not support a strong Northern Hemisphere net land carbon sink.

## INTRODUCTION

The net land carbon sink has long been considered the least certain term in the global carbon budget. A fundamental constraint on this flux is the difference between the integral of fossil fuel emissions and carbon accumulation in atmosphere and ocean reservoirs. A positive difference provides evidence of a land sink, whereas a negative difference indicates a source. The strength of this approach is tied to the relatively high accuracy and precision with which fossil fuel emissions, the atmospheric CO_2_ growth rate, and ocean uptake can be measured. One of the first applications of this constraint was undertaken by Broecker *et al.* (*1*) to evaluate the likelihood that the land was a large net source during 1955–1973 as a consequence of deforestation (*2*, *3*). Using new radiocarbon and tritium tracers from the Geochemical Ocean Sections Study to constrain rates of ocean carbon uptake with a box diffusion model (*4*), this analysis revealed that the net land carbon sink was close to neutral, indicating that deforestation carbon losses were likely smaller than initially estimated.

Drawing upon this approach to varying degrees, multiple carbon cycle assessments have concluded that the land biosphere has acted as a relatively strong net carbon sink since the 1990s when averaged on a decadal timescale. This is the consensus view from the Third, Fourth, Fifth, and Sixth IPCC Assessment Reports (*5*–*8*) and the Global Carbon Project (*9*, *10*). While fossil fuel emissions increased rapidly in the 1990s and 2000s, the atmospheric CO_2_ growth rate increased more slowly (*11*), leaving more room in the budget for accelerating carbon uptake by oceans and land (*12*). Early improvements in estimating the ocean flux using models (*13*, *14*) and analysis of repeat ocean transect observations (*15*, *16*) were critical in refining the ocean component of the global budget and constraining the magnitude of the net land flux by difference. For example, in the IPCC Third Assessment Report, the differencing approach is used to estimate a net land carbon sink of 1.4 ± 0.7 Pg C/year for the 1990s, with a reported uncertainty that is consistent with combining the uncertainties of the other budget components, assuming they are statistically independent.

Other lines of evidence emerged during the 1990s that reinforced the strong net land carbon sink paradigm. Precise measurements of multiyear global trends of atmospheric O_2_ provided an independent approach for partitioning land and ocean sinks (*17*–*19*). While the land carbon sink generates O_2_ in proportion to the stoichiometry of the organic matter that accumulates within terrestrial ecosystems (*17*, *20*, *21*), the ocean carbon sink does not generate O_2_ because it is primarily regulated by air-sea CO_2_ gas exchange, seawater carbonate chemistry, and ocean mixing that carries the anthropogenic carbon out of surface layers and into the deeper ocean. For the 1991–1994 period, when the first atmospheric O_2_ measurements became available, O_2_ consumption by fossil fuel burning was estimated to be about 44% larger than the observed rate of decline in the global atmosphere. This difference necessitates a strong net land carbon sink to balance the atmospheric O_2_ and CO_2_ budgets simultaneously (*18*).

Parallel work analyzing north-south differences in atmospheric CO_2_ provided evidence that most of the global net land carbon sink is concentrated in the Northern Hemisphere (*22*–*24*). Tans *et al.* (*22*) examined the interhemispheric CO_2_ gradient using a three-dimensional atmospheric model that carried CO_2_ as a tracer. In this study, model simulations of the interhemispheric CO_2_ gradient were found to be considerably higher than the observed gradient when the model was forced by spatially gridded fossil fuel emissions and ocean surface fluxes. Budget scenarios with a strong northern mid-latitude net land carbon sink lowered predicted atmospheric CO_2_ levels in this latitude zone, offsetting the influence of fossil fuel emissions and bringing the model into agreement with the interhemispheric gradient, surface ocean observations of dissolved CO_2_, and the global atmospheric CO_2_ growth rate. Phase 3 of the Atmospheric Transport Model Comparison Project (TransCom 3) confirmed the presence of a strong northern mid-latitude land sink during the 1990s, drawing upon a set of 16 different atmospheric models, a larger set of atmospheric observations, and an improved set of surface fluxes that served as prior constraints (*23*). Although aircraft measurements subsequently revealed that some of the atmospheric models likely had vertical mixing that was too weak, especially during Northern Hemisphere winter, the revised budget after adjusting for these biases still included a robust (but smaller) northern land sink (and a weaker tropical source) for the period of the mid-1990s (*24*).

Additional early land surface, remote sensing, and atmospheric studies yielded results that were broadly consistent with a strong net land carbon sink in the Northern Hemisphere. Eddy covariance measurements of net ecosystem CO_2_ exchange from a temperate deciduous forest provided strong evidence for substantial rates of carbon accumulation within the tower footprint (*25*). Long-term satellite time series of normalized difference vegetation index (NDVI), a measure closely related to leaf area, indicated that northern ecosystems were greening across North America and Eurasia (*26*, *27*). Multidecade increases in the amplitude of the annual cycle of atmospheric CO_2_ at Utqiaġvik (previously known as Point Barrow) and Mauna Loa indicated that the metabolism of northern ecosystems was changing at a hemispheric scale, with greater CO_2_ uptake during the summer growing season and higher levels of ecosystem respiration during fall, winter, and spring (*28*). These observations from leading research groups contributed to an early consensus that the northern terrestrial biosphere was actively accumulating carbon in response to multiple global change drivers (*5*, *29*, *30*).

Several different mechanisms have been proposed to explain global and northern net land carbon sinks, including increasing levels of atmospheric CO_2_ (*31*–*33*), increasing levels of nitrogen and phosphorus deposition (*34*–*37*), aerosol-driven changes in diffuse light (*38*–*40*), a lengthening growing season (*26*, *28*, *41*, *42*), decreases in near-surface O_3_ in some northern mid-latitude regions (*43*), and land use change (*44*, *45*). The influence of these mechanisms varies considerably in magnitude and regional expression within global land models (*46*, *47*). The increasing positive trend in atmospheric CO_2_ is widely accepted as one of the most important mechanisms at a global scale for the net land carbon sink (*48*). It is, without question, the single largest driver of carbon uptake in land surface models (*46*, *47*). While deforestation and forest degradation are critical drivers of carbon losses from tropical forest ecosystems (*49*, *50*), reforestation of previously cleared areas and afforestation are well-known mechanisms for carbon uptake in northern temperate forest ecosystems. For example, in North America, the recovery of eastern forests following a westward shift in agriculture during the 18th and 19th centuries, along with fire suppression in western forests, are well-documented processes that structure the spatial pattern, temporal evolution, and magnitude of terrestrial carbon uptake (*51*, *52*). Across China, afforestation efforts in six major ecological restoration projects over the past three decades have led to widespread increases in forest cover (*53*) and high rates of carbon accumulation (*54*), which are likely to persist in future decades (*55*).

The uncertainty in the net land carbon sink remains notably large at both global and hemispheric scales. At a global scale, contemporary estimates of this flux from O_2_ observations are considerably lower than those derived from atmospheric inversions or dynamic global vegetation models (*56*). In the Northern Hemisphere, net land carbon sink estimates from atmospheric inversions are often much higher than estimates from models (*8*, *57*, *58*). At the same time, global biomass time series derived from multiple satellite data products show that only a small fraction of the net land carbon sink accumulates within living vegetation (*59*, *60*). These diverging perspectives provide a strong motivation for developing a unifying framework. In this review, we first compare atmospheric inversion and remote sensing–derived estimates of the net land carbon sink globally and in the Northern Hemisphere, relying extensively on published approaches and datasets. Drawing on this comparison and additional synthesis, we then present evidence for a weak net land carbon sink and describe a pathway for closing the global budget that partially reconciles several key constraints on the carbon cycle. Next, we compare global models with remote sensing–derived estimates of vegetation carbon and provide suggestions for improving model agreement with a weaker land sink in future assessments. Finally, we describe several ways a weak land carbon sink hypothesis might be incorrect and several promising approaches for testing the hypothesis.

## COMPARING THE INTEGRAL OF THE LAND FLUX WITH STOCK CHANGES

With long-term continuity in atmosphere measurements and remote sensing observations of the land surface, it is becoming increasingly feasible to assess whether the time integral of net land carbon sink derived from an atmospheric inversion is consistent with the long-term observed change in the terrestrial carbon stock. This comparison, represented by Eq. 1, can be undertaken at regional, continental, hemispheric, or global scales

$$\int F_{\text{LAND}}\left(t\right)\cdot \mathrm{dt}=∆C_{\text{LAND}}$$ (1)

where $∆C_{\text{LAND}}$ is the long-term change in the land carbon stock, and *F*_LAND_ in year *t* is the net land carbon sink associated with terrestrial ecosystem responses to changing atmospheric composition and climate (*S*_LAND_) after subtracting land use change emissions (*E*_LUC_); see Supplementary Text for more information. To be complete, *F*_LAND_ should include both land-atmosphere and lateral components, and Δ*C*_LAND_ should include aquatic and urban ecosystems, although, in practice, it is challenging to measure all of these terms simultaneously due to a lack of data. For this review, we also decompose the change in the land carbon stock, Δ*C*_LAND_, into components associated with living vegetation (including above and belowground components), Δ*C*_VEG_, fine and coarse litter (including dead wood), Δ*C*_LITTER_, and soil organic matter, Δ*C*_SOIL._

$$∆C_{\text{LAND}}=∆C_{\text{VEG}}+∆C_{\text{LITTER}}+∆C_{\text{SOIL}}$$ (2)

These ecosystem pools also map directly onto available simulation output from Earth system models participating in the Coupled Model Intercomparison Project (CMIP) (*61*), enabling a direct comparison between models and observations.

**Fig. 1.**
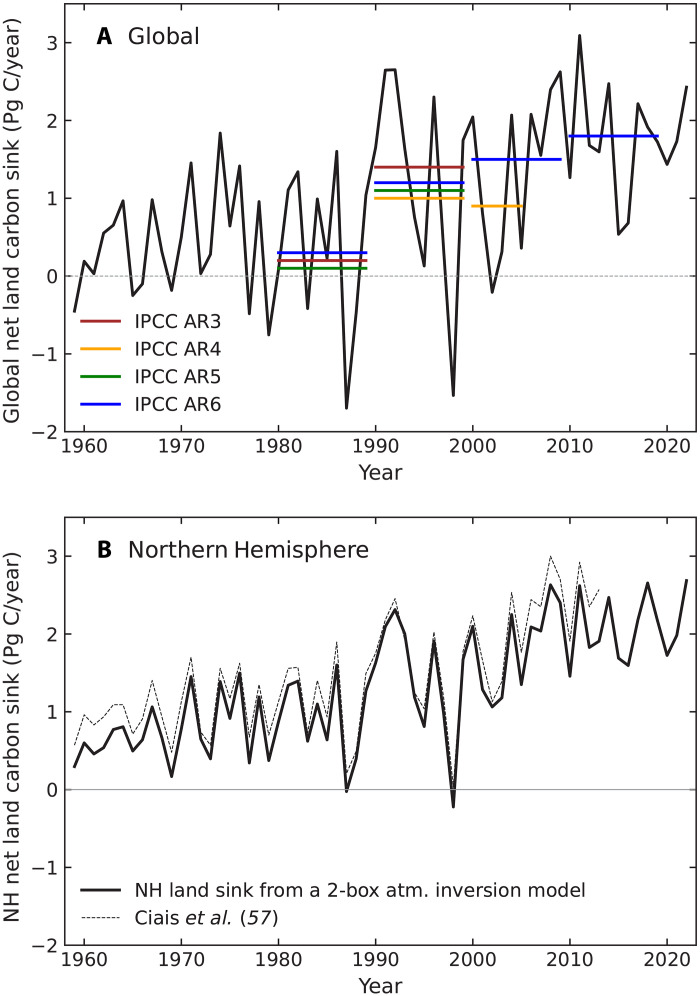
Global and Northern Hemisphere net land carbon sinks. (**A**) The global net land carbon sink from the 2023 Global Carbon Project budget from 1959 to 2022 is shown with a solid black line, alongside multiyear means reported by the Intergovernmental Panel on Climate Change. (**B**) The Northern Hemisphere (NH) net land carbon sink derived from a two-box atmospheric inversion model (*57*) over the same period. The original Ciais *et al.* (*57*) time series for 1959–2013 is shown in (B) with the gray dotted line. The global cumulative sum from 1959 to 2022, as shown in (A), is about 57 Pg C, whereas for the Northern Hemisphere shown in (B), the cumulative sum is about 85 Pg C.

As an initial exercise to understand the magnitude of expected stock changes, we focus on global and Northern Hemisphere spatial domains and the time integral from 1959 to 2022. For illustration, as a potential upper bound, we assess the relative change in *C*_VEG_ if the carbon accumulation associated with the net land sink were to reside solely in this pool. At a global scale, the 1959–2022 integral of the net land flux reported in the 2023 Global Carbon Budget (*56*) yields a cumulative change of 57 Pg C (Fig. 1A). Near the end of this record, in 2019, a global estimate of *C*_VEG_ derived from remote sensing and inventory observations is about 411 Pg C (*49*, *59*). Combining these two estimates, if all the net land flux accumulates in vegetation carbon, we expect about a 16% increase in the size of this global pool over the 64-year interval. Given uncertainties, this moderate-sized change would be challenging to detect.

To repeat this analysis for the Northern Hemisphere, we extended the two-box atmospheric inversion methodology from Ciais *et al.* (*57*) to estimate the northern net land carbon sink from 1959 to 2022 (Fig. 1B). For this analysis, we used the CO_2_ time series at Mauna Loa and the South Pole from the Scripps Institution of Oceanography CO_2_ Program (*62*, *63*), the 2023 Global Carbon Project budget for the time series of fossil fuel emissions and the magnitude of global land and ocean sinks (*56*), and the data-constrained Ocean Circulation Inverse Model [OCIM; (*64*)] to distribute the anthropogenic ocean sink in Northern and Southern Hemispheres (see Supplementary Text for more information). Interannual and decadal variability from our two-box model closely matched the earlier estimates from Ciais *et al.* (*57*), but the mean net carbon sink magnitude was about 18% smaller during the overlap period from 1959 to 2013 (Fig. 1B). The two-box model suggests that the northern net land carbon sink has more than doubled over the past five decades, increasing from about 0.9 Pg C/year in the 1980s to 1.4 Pg C/year in the 1990s, 1.8 Pg C/year in the 2000s, and 2.1 Pg C/year in the 2010s. Integrating this flux from the beginning of the Mauna Loa and South Pole time series, we obtain an accumulation of northern land carbon of 85 Pg C. At the same time, the vegetation carbon stock in the Northern Hemisphere (about 262 Pg C) is considerably smaller than the global total. Combining these two values, if all of the Northern Hemisphere land flux accumulated in vegetation carbon, we would expect to see about a 48% increase in this stock. Of course, carbon would also likely accumulate in litter, soil, and other reservoirs (Eq. 2), lowering the relative change expected for Δ*C*_VEG_. Nevertheless, this magnitude of change might be helpful in comparison with expert opinion from researchers in forestry, ecology, and carbon cycle science communities. Over a human lifetime, one would expect a hemispheric change of this magnitude to be noticeable, pervasive, and detectable in field datasets, such as long-term inventory plots.

A more quantitative comparison of atmospheric inversion and remote sensing–derived estimates of land carbon can be made over the past several decades, as increases in data availability and quality have reduced uncertainties in both approaches. Here, we focus on the 2000–2019 period, during which time satellite data products from Terra, Aqua, and ICESat have enabled the global-scale tracking of changes in vegetation biomass. For a remote sensing–derived estimate of Δ*C*_VEG_, we merged biomass time series developed by the Jet Propulsion Laboratory (JPL) (*59*) and Chloris (*49*). These remote sensing products are derived by combining information from forest inventory observations, space-based lidar measurements of forest structure, and wall-to-wall daily coverage of surface reflectance and temperature from the Moderate Resolution Imaging Spectroradiometer sensor on NASA’s Aqua and Terra satellites. They exhibit trends comparable to those derived from observations of vegetation optical depth (*65*).

To compare the inversion and remote sensing–derived estimates of carbon accumulation within terrestrial ecosystems (i.e., Δ*C*_LAND_), we developed a simple approach for scaling up the remote sensing–derived estimates of Δ*C*_VEG_ to Δ*C*_LAND_

$$∆C_{\text{LAND}}=S\cdot ∆C_{\text{VEG}}$$ (3)

Specifically, we estimated a scale factor, *S*, from CMIP6 models (table S1) as the ratio of total carbon accumulation within an ecosystem to the accumulation within living vegetation

$$S=\frac{∆C_{\text{VEG}}+∆C_{\text{LITTER}}+∆C_{\text{SOIL}}}{∆C_{\text{VEG}}}=\frac{\sum \text{NBP}}{∆C_{\text{VEG}}}$$ (4)

For each model, we estimated the numerator in Eq. 4 as the global sum of the net biome production (NBP) variable from the midpoint of 2000 to the midpoint of 2019. For the denominator, we used the long-term change in vegetation carbon, Δ*C*_VEG_, for each model over the same domain and period. From this analysis, we obtain a value for *S* of 1.6 ± 0.6 (±1σ) by averaging the *S* values across models. This approach assumes that changes to ecosystem carbon stocks originate initially from the influence of global change drivers on inputs and losses from the vegetation carbon pool, with downstream adjustments to this perturbation in litter and soil carbon reservoirs. Although this type of scaling approach may break down on longer timescales in the future, for example, as losses from permafrost carbon become decoupled from changes in forest growth (*66*), it is broadly consistent with the ways we expect carbon to flow through ecosystems in response to the primary mechanisms identified as the most critical drivers of the contemporary land carbon budget, including CO_2_ fertilization and land use change. Consistent with an input-driven sink, model-to-model variation in Δ*C*_VEG_ accounts for 36% of the variance in cumulative NBP across CMIP6 models during 2000–2019 (*P* = 0.001), as shown in table S1. In addition, Δ*C*_VEG_ explains an average of 70 ± 20% of the within-model spatial variability of cumulative NBP. In computing *S* in this way, we draw upon information from the models’ internal partitioning of carbon flows into downstream pools and their associated turnover times, without directly using the models’ NBP estimates. We note that this scaling factor may represent an upper bound for accumulation rates in detrital pools; for example, syntheses of forest inventory observations yield considerably lower estimates of *S* in many regions. Using data from forest inventories reported by Pan *et al.* (*67*) from their extended data table 3 and considering detrital and product pools, *S* values range from about 1.25 in tropical forests to about 1.45 for temperate forests. One notable exception to this range is the boreal forests in Russia, where long-term carbon accumulation, as reported for deadwood, litter, and soils, far exceeds the magnitude of decadal-scale changes in living biomass (*67*).

**Fig. 2.**
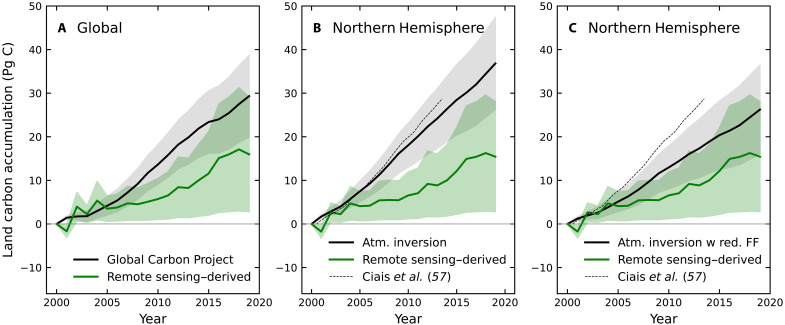
Net land carbon accumulation derived from atmospheric inversion and remote sensing approaches. (**A**) Estimates for the global land surface. (**B** and **C**) Estimates for the Northern Hemisphere. In (A), the Global Carbon Project net land sink estimate is derived from the difference between the sum of fossil fuel emissions and accumulation rates in atmospheric and ocean reservoirs, as reported in the 2023 budget. The remote sensing–derived estimate is derived by multiplying a fused estimate of Δ*C*_VEG_ from JPL and Chloris biomass products by a scale factor, *S*, to obtain cumulative NBP. In (B), the two-box atmospheric inversion estimate is derived from the time series shown in Fig. 1B. The remote sensing–derived estimate in (B) is nearly the same as in (A) because almost all of the observed carbon gain over this period occurs in the Northern Hemisphere. Atmospheric inversion estimate for the Northern Hemisphere net land carbon sink for a budget scenario in which the fossil fuel (FF) flux has been reduced by 6% and the ocean flux has been increased by 8% relative to the Global Carbon Project baseline. A weaker fossil fuel flux generates a weaker north-south CO_2_ gradient in the two-box atmospheric inversion, allowing for closer agreement of the inferred northern land sink from the inversion with the estimate derived from vegetation carbon accumulation.

Applying *S* to the remote sensing–derived estimate of Δ*C*_VEG_ and accounting for uncertainties in both terms, we obtain an estimate of global carbon accumulation on land, Δ*C*_LAND_, of 16.0 ± 13.1 Pg C from the midpoint of 2000 to the midpoint of 2019 (Fig. 2A). Converting this to an annual net land carbon sink (*F*_LAND_) yields an estimate of 0.8 ± 0.7 Pg C/year for 2000–2019. Over this same interval, the Global Carbon Project mean of 1.6 ± 0.6 Pg C/year is nearly double the remote sensing–derived estimate.

In the Northern Hemisphere, carbon accumulation on land derived from the two-box atmospheric inversion is about 2.4 times larger than the remote sensing–derived estimate (Fig. 2B). Expressed as an annual flux, the remote sensing–derived estimate for the Northern Hemisphere is nearly the same as the global-scale flux, with a 2000–2019 mean of 0.8 ± 0.7 Pg C/year because nearly all of the carbon accumulation in this estimate occurs in northern ecosystems. However, the two-box atmospheric inversion estimate of the northern land sink is significantly higher, with a mean of 1.9 ± 0.5 Pg C/year. Although the uncertainties remain substantial, the difference in the predicted carbon accumulation rate within terrestrial ecosystems between the atmospheric inversion and remote sensing–derived approaches represents a fundamental gap in our understanding of the global carbon cycle. This difference is particularly pronounced in the Northern Hemisphere, where top-down atmospheric constraints on the carbon cycle indicate that the net land carbon sink is growing decade by decade, even as the impacts of drought and fire have intensified.

## THE WEAK LAND CARBON SINK HYPOTHESIS

### Hypothesis statement

We hypothesize that the remote sensing–derived estimate of the global net land carbon sink is the most accurate, with a global mean of 0.8 ± 0.7 Pg C/year for the period 2000–2019 and a distribution that is predominantly concentrated in the Northern Hemisphere. We make this assertion because this approach relies extensively on direct measurements of the land surface. A global net land sink of this magnitude is about 46% lower than the 2023 Global Carbon Project estimate for the same period (Table 1). Many current approaches for estimating the net land sink rely explicitly or implicitly on precise and unbiased fossil fuel emission inventories and ocean flux estimates. Even a small bias in fossil fuel emission inventory can considerably affect net land carbon sink estimates when the land term is estimated as the difference between other budget components. At the same time, information about the magnitude of this residual “target” for land may leak into other assessment methods, such as dynamic global vegetation models, through parameter choices during model development cycles. This introduces the potential for some convergence (and circularity) in land sink estimates derived from different approaches, as they may not be entirely independent of one another.

**Table 1.** Consensus and proposed global carbon budgets for 2000–2019. The consensus estimate is from the 2023 budget of the Global Carbon Project. The weak net land carbon sink hypothesis budget is described in the main text.

Flux component: Units: Pg C/year	Global Carbon Project	Weak land sink hypothesis	Percent difference
	Mean ± 1 σ	Mean ± 1 σ	
Fossil fuel emissions	8.6 ± 0.4	8.1 ± 0.9	−6
Atmospheric growth rate	4.6 ± 0.1	4.6 ± 0.1	0
Ocean sink	2.5 ± 0.4	2.7 ± 0.5	8
Land sink	1.6 ± 0.6	0.8 ± 0.7	−46

For the weak net land carbon sink hypothesis to be viable, we need to (i) provide a pathway for closing the global budget with adjustments to other terms, (ii) reconcile the weak land sink with global constraints provided by the interhemispheric CO_2_ gradient and long-term atmospheric O_2_ trends, and (iii) achieve consistency with other atmosphere and land surface observations.

### Closing the budget with adjustments to the ocean sink and fossil fuel emissions

To close the global carbon budget with a weak net land sink, we must address a remaining imbalance of about 0.7 Pg C/year (Table 1). To zero out this imbalance, we propose increasing the ocean sink estimate by 8% (about 0.2 Pg C/year) and decreasing the fossil fuel emission estimate by 6% (about 0.5 Pg C/year) for the period 2000–2019, relative to the 2023 budget from the Global Carbon Project (Table 1). We describe below evidence for why these changes are plausible.

The consensus estimate of the ocean carbon sink from the Global Carbon Project depends on estimates derived from (i) three-dimensional ocean models forced by the observed time series of atmospheric CO_2_ and (ii) models of air-sea gas exchange forced by observations of ocean-atmosphere differences in the partial pressure of CO_2_. Within this set of estimates, the three-dimensional ocean models occupy the lower bound of the reported sink estimates (*56*). Two recent studies suggest that many ocean models underestimate anthropogenic carbon uptake relative to available data constraints. Fu *et al.* (*68*) demonstrate that CMIP5 and CMIP6 ocean models significantly underestimate a data-constrained estimate of the ocean anthropogenic carbon inventory change from 1994 to 2007 (*69*). Terhaar *et al.* (*70*) show, using an emergent constraint approach, that three ocean model properties, including the strength of the Atlantic meridional overturning circulation, the surface Revelle factor, and sea surface salinity in the Southern Ocean, explain a considerable amount of the model-to-model variance in anthropogenic carbon uptake across different CMIP6 models. Using observations of these different ocean properties, they derive an unbiased estimate that is about 9% higher than that predicted by the original models. Here, we applied a simple correction to account for a potential low bias in contemporary ocean models. Specifically, we increase the magnitude of the Global Carbon Project 2023 ocean carbon sink time series by about 8% in our new budget so that the mean flux during 1994–2007 matches the data-constrained estimate of anthropogenic carbon for this period (*69*), after making a small adjustment to account for the natural loss component driven by climate warming (*68*). Our data-constrained adjustment increases the magnitude of the ocean sink from 2.5 ± 0.4 Pg C/year (the Global Carbon Project historical central estimate during 2000–2019) to 2.7 ± 0.5 Pg C/year. We also propose that it is plausible to increase the relative uncertainty for this estimate from the 14% shown in figure 2 of the 2023 Global Carbon Project report (*56*) to 20% to reflect the remaining challenges in accurately simulating ocean circulation and reconciling air-sea gas exchange, O_2_/N_2_, and three-dimensional ocean modeling approaches.

Given that the carbon accumulation rate in the atmosphere is well known, we propose reducing fossil fuel emissions by 6% from 2000 to 2019 as a final step to close the global budget (Table 1). In the past, when the net land sink was estimated by difference, its uncertainties were often calculated by adding, in quadrature, the uncertainties of the other better-known budget terms. For this hypothetical budget, we take the reverse approach, computing the uncertainty for the fossil fuel flux by using the uncertainties from the other terms, which yields an estimate of ±11%. This uncertainty range is about a factor of 2 higher than the 2023 estimate reported by the Global Carbon Project. With these adjustments to the mean and uncertainty range, we obtain a 2000–2019 net fossil fuel flux of 8.1 ± 0.9 Pg C/year (Table 1).

Most of the information used in contemporary fossil fuel emission inventories originates from individual countries’ self-reporting of their energy use activity and emission factor data to the International Energy Agency and the United Nations (*71*). A potential positive bias in emission reporting might arise because of political pressure to project strong economic growth. Evidence for overreporting of gross domestic product (GDP) has been documented for several countries (*72*–*75*). At a state or province level, pressure for companies to overreport earnings may occur, for example, if the promotion of local politicians is tied to success in meeting GDP performance targets (*76*). At a national level, GDP might be overstated to benefit a ruling political party, strengthen currency valuation, downplay the effects of a recession or sanctions, attract international investment (*77*), or enhance political capital during trade negotiations (*78*). A comparison of GDP growth with increases in nighttime light detected by satellites provides indirect evidence of the overreporting of GDP in some autocratic countries (*79*).

GDP and energy use are closely linked: Greater economic growth typically enables higher levels of energy consumption (*80*), while energy supply is a key driver of productivity in sectors such as manufacturing (*81*). The strong coupling between GDP, energy use, and carbon emissions—captured by the Kaya Identity (*82*)—raises the possibility that, in scenarios where GDP estimates are inflated, energy use statistics might also be adjusted to support the financial narrative. In many, although not all, countries, GDP and energy data are compiled by a single government agency, whose degree of political independence can vary. In China, reported consumption of natural gas, coal, and oil is often considerably higher at a provincial level than at the national level, likely reflecting political pressure on local authorities to make their energy use statistics align with their GDP growth reports (*83*). Although supported by less direct evidence, another potential political incentive for inflating emission data could stem from international climate negotiations under the United Nations Framework Convention on Climate Change (UNFCCC), established in 1992. By overstating emissions before the imposition of binding reductions, a country could establish a higher baseline, thereby easing the path to meeting future targets and gaining economic advantage. In contrast, current political incentives may now favor reporting successful emission reductions to signal climate leadership and progress toward international commitments, especially in the post-2015 era of the Paris Agreement and the reporting of nationally determined contributions. While most national energy and emission reporting is likely free from political bias, it is essential to recognize that this component of the carbon budget is constructed in a fundamentally different manner than others, particularly in terms of the application of the scientific method, data transparency, and reproducibility. In addition to potential political influences, unintentional biases may also emerge from incomplete sampling or methodological limitations in specific sectors or regions (*84*).

**Fig. 3.**
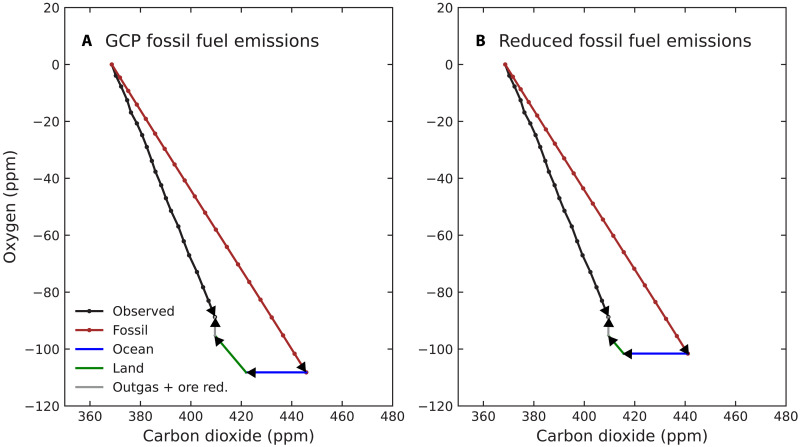
Vector diagrams showing land and ocean carbon sinks estimated from atmospheric O_2_ and CO_2_ trends. (**A**) The land and ocean sinks calculated using the Global Carbon Project’s 2023 fossil fuel emission time series. (**B**) The land and ocean sinks obtained using the fossil fuel emission time series that has been reduced by 6%. This scenario yields a smaller fossil fuel vector, a net land sink that is similar to the remote sensing–derived estimate, and an ocean sink that is nearly the same as the OCIM estimate described in the main text. The atmospheric observations in both panels are from the Scripps Institution of Oceanography Oxygen Program (*92*, *93*). The analysis spans the period from mid-2000 to mid-2019. ppm, parts per million.

Previous work drawing upon atmospheric observations provides additional evidence for a positive bias in fossil fuel emissions. Francey *et al.* (*11*) were the first to report that the global fossil fuel emission time series may not be entirely consistent with atmospheric constraints. They show that increases in global fossil fuel and land use emissions between 1990 and 2010 did not generate the expected concurrent increases in the atmospheric CO_2_ growth rate, particularly for the period between 2001 and 2010 when fossil fuel emissions alone rapidly increased by more than 2 Pg C/year [as shown in figure 3 of (*11*)]. Saeki and Patra (*85*) provide atmospheric inversion evidence indicating a high bias in fossil fuel emission inventories for East Asia. Their analysis was initially motivated by a study of atmospheric methane, which found that the 2002–2012 increase in methane emissions inferred from a GLOBALVIEW-based inversion—independently supported by a multiyear aircraft transect record—was substantially smaller for the region than the trend inferred from coal and gas emission inventories (*86*). When the authors applied a proportional reduction to CO_2_ emissions from these sectors, the resulting 41% decrease in fossil fuel emission growth led to a substantially weaker net land carbon sink in the inversion, which was in closer agreement with regional estimates from TRENDY land models (*85*). Notably, the magnitude of this East Asia–specific fossil fuel adjustment alone is comparable in magnitude to the global correction needed to reconcile the carbon budget with a weak net land carbon sink.

### Reconciling the weak land sink with the interhemispheric CO_2_ gradient and O_2_ trends

A revised budget with a weak net land carbon sink and a smaller fossil fuel flux offers several advantages for reconciling the global budget with the interhemispheric CO_2_ gradient and long-term trends in atmospheric O_2_. For atmospheric inversions, the 0.5 Pg C/year reduction in fossil fuel emissions considerably weakens the need for a strong Northern Hemisphere land sink, as it reduces the north-south interhemispheric CO_2_ gradient in atmospheric transport model simulations. In the two-box atmospheric model, decreasing the fossil fuel emission flux by 6% (and increasing the ocean flux by 8%) causes the 2000–2019 Northern Hemisphere net land carbon sink to decrease from 1.9 ± 0.5 Pg C/year to 1.4 ± 0.5 Pg C/year (Fig. 2C). This adjustment to the land sink helps bring the atmospheric inversion estimate into closer agreement with the remote sensing–derived estimate.

Some of the remaining difference between the atmospheric inversion and remote sensing–derived estimates in the Northern Hemisphere may be partly explained by carbon flows from managed and unmanaged ecosystems into urban areas (*67*). Specifically, carbon accumulates in cities in the form of paper, wood, and fiber products within homes and buildings (*87*, *88*), as well as organic debris within landfills (*83*, *84*), and may constitute a flux of ~0.2 Pg C/year over the past several decades (*60*, *67*). In addition, carbon builds up in both aboveground and belowground carbon pools in urban forests and parks (*89*, *90*). These carbon flows into urban areas and other forms of intensifying land use are also likely to accelerate aquatic dissolved organic and inorganic carbon flows to the oceans (*91*). A critical next step is to develop a comprehensive assessment of carbon stock changes in urban areas that accounts for building, landfill, urban ecosystem, and other infrastructure components.

A smaller fossil fuel flux also considerably reduces the need for a global net land carbon sink when simultaneously closing global O_2_ and CO_2_ atmospheric budgets (Fig. 3). Using O_2_ and CO_2_ measurements from Alert, Mauna Loa, Cape Grim, and South Pole stations in the Scripps Institution of Oceanography Global Oxygen Program (*18*, *92*, *93*), we performed land and ocean net land sink partitioning for two cases (see the Supplementary Materials). The first case uses the Global Carbon Project’s net fossil fuel emission inventory, and the second case maintains all parameters unchanged while reducing the fossil fuel flux by 6%. For the first case, the net land sink from 2000 to 2019 is 1.4 ± 0.7 Pg C/year, and the ocean sink is 2.7 ± 0.3 Pg C/year. The first case yields a net land sink that is somewhat lower than the Global Carbon Project’s estimate obtained by combining dynamic global vegetation and land use models (1.7 ± 0.9 Pg C/year) but is within the reported uncertainty range (Fig. 3A). This analysis is also broadly consistent with the O_2_-derived land/ocean sink partitioning shown in figure 12 of (*56*) for the 2013–2022 period.

For the second case, when fossil emissions are reduced by 6%, the land sink decreases to 0.7 ± 0.6 Pg C/year, and the ocean sink increases to 2.9 ± 0.3 Pg C/year. This case provides a budget that is more consistent with the remote sensing–derived estimate of the net land carbon sink and generates an ocean sink that is nearly identical to the data-constrained estimate from OCIM. Thus, with a relatively small downward adjustment to the fossil fuel emission inventory, many available atmosphere and land remote sensing–based estimates of the land sink become more closely aligned.

### Consistency with other land and atmosphere observations

As described in Introduction, other influential direct and indirect observational constraints on land carbon cycling include eddy covariance measurements of net ecosystem exchange (NEE), satellite-derived greening trends, and multidecadal changes in the amplitude of the CO_2_ annual cycle in the Northern Hemisphere. Here, we revisit these constraints in the context of the weak land carbon sink hypothesis. Forest inventory observations are also a potentially valuable constraint; however, heavily restricted access to site-level plot information, a lack of transparency in scaling methods, and limited spatial coverage make them unsuitable for use as an independent verification at present. We further discuss forest inventory measurements in the section on Ways to test the hypothesis.

From early studies in agricultural and forest ecosystems (*25*, *94*) to today’s global network of hundreds of sites (*95*), eddy covariance tower measurements have revolutionized our understanding of ecosystem energy, water, and carbon fluxes across timescales from hours to decades. This network, coupled with standardized data processing, has been instrumental in improving land surface model performance. While the network was initially motivated, in part, by interest in long-term terrestrial carbon sequestration, challenges such as nocturnal cold air drainage in forests remain (*96*–*98*). If unaccounted for, this can lead to underestimates of nighttime respiration (*99*) and overestimates of the long-term integral of NEE flux into the land surface. For example, machine learning models trained on FLUXNET data estimate a global NEE flux of ~17 Pg C/year from 1982 to 2009 (*100*). Roughly one-third to one-half of this flux is offset by carbon returned to the atmosphere via fire, methane, and volatile organic compound emissions, and respiration linked to lateral flows through aquatic systems, agriculture, or harvested wood products. The fate of the remaining flux is unclear, potentially reflecting systematic sampling and measurement biases (*101*), which complicates use of the network for refining net land sink estimates at the time and space scale addressed in this review.

Multiple lines of evidence indicate that global gross primary production (GPP) is increasing. This trend is supported by eddy covariance tower observations (*102*, *103*), satellite-derived vegetation index and leaf area time series (*26*, *104*–*106*), light use efficiency and prognostic ecosystem models (*107*–*109*), firn measurements of carbonyl sulfide (*110*), and changes in the amplitude and shape of the Northern Hemisphere atmospheric CO_2_ seasonal cycle (*28*, *111*–*114*). There are at least three different mechanisms that can explain why multidecadal increases in GPP might not directly translate into high rates of net land carbon accumulation. First, increases in GPP often do not yield proportional increases in net primary production (NPP), and NPP itself can become further decoupled from long-term carbon storage in response to elevated CO_2_ and other global change drivers (*115*–*117*). Second, rates of disturbance and background tree mortality are increasing in many forest ecosystems (*118*–*120*), reducing carbon residence times. Third, rates of carbon storage in soils are likely limited by microbial feedbacks (*121*) and the availability of mineral binding sites (*122*). These mechanisms are further explored in the following section in the context of improving agreement between a budget with a weak land carbon sink and estimates from land models.

In northern temperate and boreal ecosystems, the increasing frequency of wildfires and agricultural intensification likely contribute to increases in GPP and the amplitude of the annual cycle of CO_2_ without a close coupling to long-term carbon storage. Increases in annual burned area cause a shift in the stand age distribution of forests, allowing deciduous shrub and tree species to become prevalent at a landscape scale (*123*). These plant functional types exhibit much higher rates of mid-summer photosynthesis than evergreen needleleaf trees (*123*–*125*). At the same time, increasing fire activity is also reducing carbon stocks in soil organic matter layers (*126*), making it difficult to assess the overall impact of the changing disturbance regime on the net ecosystem carbon balance. Agricultural expansion and intensification have similarly increased mid-season CO_2_ drawdown without a strong link to carbon storage (*127*, *128*).

### Compatibility with uncertainty estimates

A weaker net land carbon sink, combined with lower fossil fuel emissions, produces an internally consistent carbon budget that aligns with constraints from satellite-derived biomass trends, the interhemispheric CO_2_ gradient, and long-term global O_2_ and CO_2_ records. This proposed shift from a strong to a weak land sink can be accommodated by relatively minor adjustments to other budget terms, remaining within the 1-sigma prior uncertainty estimates for the ocean sink (±14%) and near the 1-sigma prior uncertainty for fossil fuel emissions (±5%) reported by the Global Carbon Project as shown in Table 1. In this context, we note that it is impossible to balance a weak net land sink inferred from the satellite-derived biomass trends solely by increasing the ocean sink. While this adjustment might account for the difference between fossil fuel emissions and the atmospheric CO_2_ growth rate, it would fail in several other ways. First, it would not explain the global O_2_ record. Specifically, the resulting carbon budget would not produce enough oxygen to account for the observed long-term decline in atmospheric O_2_, which is much smaller than expected from fossil fuel combustion. Second, because the ocean carbon sink is somewhat larger in the Southern Hemisphere than in the Northern Hemisphere (*129*), strengthening it would not resolve the discrepancy between the remote sensing–derived and atmospheric inversion estimates of the Northern Hemisphere net land carbon sink that is tied to the north-south atmospheric CO_2_ gradient (Fig. 2).

## IMPROVING AGREEMENT WITH LAND MODELS

Land surface models play a crucial role in carbon cycle assessments. For example, TRENDY models (*130*) are the primary tool for quantifying the gross land carbon sink originating from processes other than land use change (i.e., *S*_LAND_ as defined in Supplementary Text) in the budgets developed by the Global Carbon Project. The ensemble of these models, when combined with a different set of land use change estimates, yields an estimate of *F*_LAND_ (1.7 ± 0.9 Pg C/year) during 2000–2019 that is nearly identical to the estimate derived from the difference between net fossil fuel emissions and fluxes into atmosphere and ocean reservoirs (1.6 ± 0.6 Pg C/year) for the 2023 Global Carbon Project budget (*56*). Here, we evaluate 17 CMIP6 models that reported *C*_VEG_ and NBP as a representative set of land models (table S1). First, we compare model estimates with the remote sensing–derived time series of *C*_VEG_ described above. In a second step, we identify a set of mechanisms that, if better represented within current models, might improve agreement with a weak net land carbon sink.

### Model evaluation

**Fig. 4.**
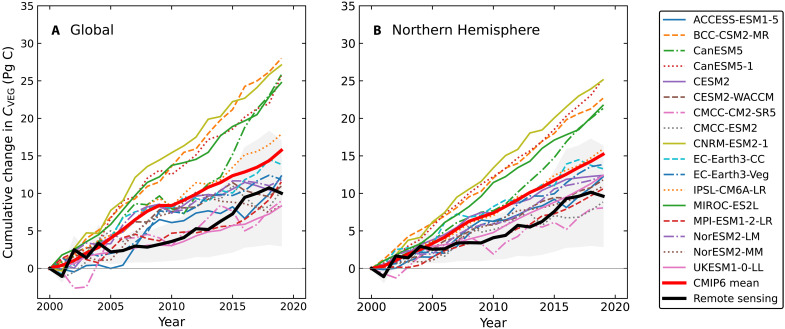
Carbon accumulation in living vegetation according to CMIP6 models and the estimate derived from satellite remote sensing. (**A**) Accumulation for the global land surface. (**B**) Accumulation for the Northern Hemisphere. These estimates encompass both aboveground and belowground carbon in living biomass and represent the total across all plant functional types.

At a continental scale, the ensemble mean from CMIP6 matches the spatial pattern of the remote sensing–derived estimate reasonably well during 2000–2019 (fig. S1), with global *C*_VEG_ from the multimodel mean (478 ± 86 Pg C) having a slight positive bias of about 16%. However, model estimates of contemporary vegetation biomass accumulation are, on average, considerably higher than those derived from remote sensing and vary substantially from model to model. The global CMIP6 multimodel mean of 15.7 ± 7.4 Pg C for Δ*C*_VEG_ is about 55% higher than the remote sensing–derived estimate of 10.1 ± 7.0 Pg C during this period (Fig. 4A). The model − remote sensing product difference is similar in the Northern Hemisphere, where the CMIP6 multimodel mean is 15.2 ± 5.5 Pg C and the remote sensing–derived estimate is 9.6 ± 6.7 Pg C (Fig. 4B). A threefold variation across models highlights the wide range of possible outcomes driven by differences in climate forcing and how key processes—photosynthesis, allocation, growth, mortality, disturbance, and land use change—are represented and parameterized.

**Fig. 5.**
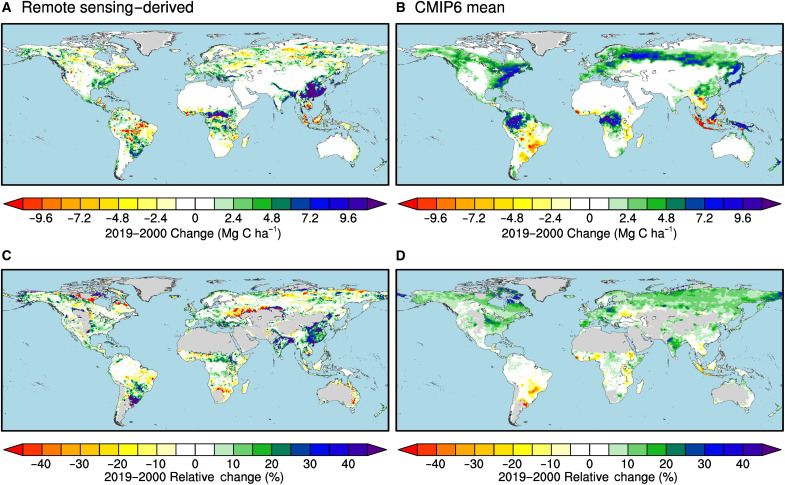
Spatial distribution of vegetation carbon trends from 2000–2019. Absolute changes are shown for the remote sensing–derived estimate of Δ*C*_VEG_ in (**A**) and the multimodel mean from CMIP6 in (**B**). The units are megagrams of carbon per hectare change over 20 years. (**C** and **D**) Cumulative percent changes over the same period. For (C) and (D), relative trends have been masked out in places where the observed *C*_VEG_ was less than 1 Mg C ha^−1^.

The spatial pattern of biomass accumulation is also fundamentally different between the models and the remote sensing–derived estimates (Fig. 5). In the remote sensing–derived estimate, high biomass accumulation rates are confined to just a few regions, including eastern Asia, northern tropical Africa, eastern North America, and some areas of temperate Southern Hemisphere South America (Fig. 5, A and C). In contrast, CMIP6 models exhibit widespread increases on all major continents, with a high level of consistency in the directionality of the response among models. The widespread pattern of increasing *C*_VEG_ from CMIP6 (Fig. 5, B and D) is consistent with a strong global physiological response of plants to rising CO_2_, as well as some uniformity in downstream allocation and mortality parameterizations across models that allow this carbon to accumulate within living vegetation. The model–satellite product difference in the magnitude and spatial continuity of biomass trends, as shown in Fig. 5, is consistent with previous work that documents similar differences for NPP (*131*).

Regional studies also indicate that Earth system models tend to predict higher biomass accumulation rates than estimates derived from remote sensing. In western boreal North America, for example, the CMIP6 multimodel mean rate of aboveground biomass accumulation is about a factor of 3 higher than a remote sensing estimate derived from Geoscience Laser Altimeter System lidar observations, Landsat imagery, and plot-level inventory observations for the 31 years from 1984 to 2014 (*132*). For temperate North America, projected increases in biomass accumulation from CMIP6 models far exceed estimates from inventory-constrained growth-mortality and climate-driven machine learning models (*133*). In both of these studies, model–remote sensing differences were attributed, in part, to an intensification of the fire regime that so far has been challenging to reproduce within models.

The positive bias in Δ*C*_VEG_ in many CMIP6 models leads directly to a positive bias in cumulative NBP, reflecting the strong causal link between these two variables in the models. The CMIP6 multimodel mean global net land sink is 1.2 ± 0.6 Pg C/year for 2000–2019—about 0.4 Pg C/year higher than the remote sensing–derived estimate, with the SD representing intermodel spread. In the Northern Hemisphere, CMIP6 models simulate a mean sink of 1.0 ± 0.4 Pg C/year, which exceeds the remote sensing estimate by about 0.2 Pg C/year but falls short of the atmospheric inversion estimate by 0.9 Pg C/year. This low bias in the models relative to the inversion estimate is consistent with previous comparisons, including between inversions and TRENDY v4 models (*57*) and between other state-of-the-art inversions and CMIP land models in the IPCC 6th Assessment (*8*).

A key finding from our analysis is the substantial spread in model outputs: Some align with remote sensing estimates for Δ*C*_VEG_ and Δ*C*_LAND_, while others are two to three times higher. A similarly wide range appears in the TRENDY dynamic vegetation model ensemble for the 2013–2022 period, as reported in the 2023 Global Carbon Project budget (*56*). The Global Carbon Project and IPCC currently average all models equally, in part due to the challenge of validating individual models. Reducing this uncertainty will require more systematic benchmarking, including the use of new Δ*C*_VEG_ time series for ensemble weighting and emergent constraint analysis.

### Strengthening the representation of key mechanisms

Many land models need lower carbon accumulation rates in vegetation, litter, and soil organic matter pools to match the magnitude of the weak land carbon sink derived from remote sensing observations. We focus our discussion here on three mechanisms that have the potential to slow carbon accumulation rates if their process-level representation were to be strengthened within the current generation of land surface models. In proposing these areas for model improvement, we are not advocating for increasing model complexity. Instead, we suggest that rebalancing model code to better represent carbon flows downstream from GPP and the influence of accelerating disturbance rates in many biomes might improve agreement with a smaller magnitude net land carbon sink.

#### Decoupling photosynthesis and growth responses to global change drivers

**Fig. 6.**
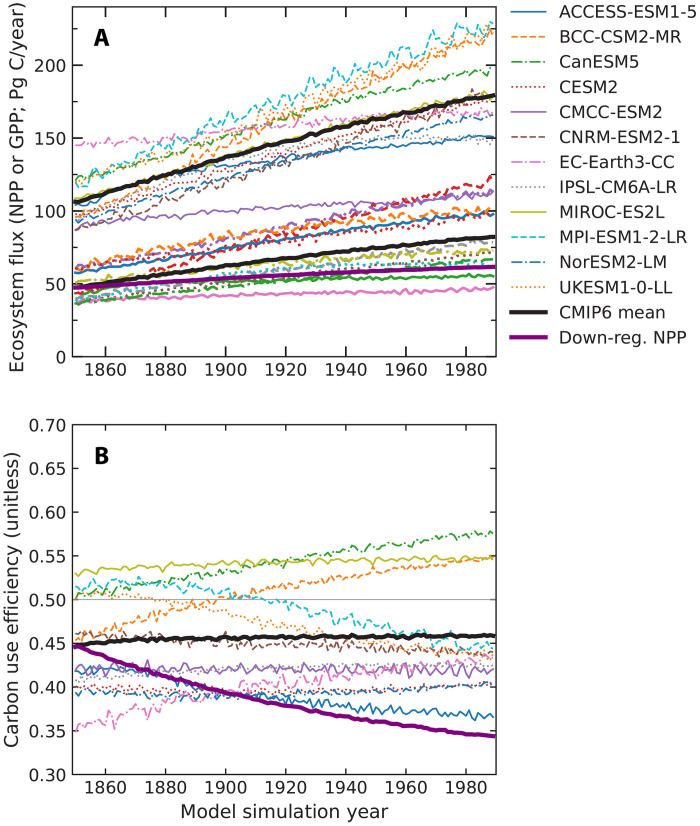
GPP and NPP responses to increasing atmospheric CO_2_ in CMIP6 models. (**A**) Individual model time series for GPP (upper set of lines) and NPP (lower set) for the idealized 1pctCO2-bgc simulation from CMIP6, in which atmospheric CO_2_ increases by 1%/year for 140 years for the biogeochemistry components of the Earth system model but remains constant at preindustrial levels in the radiative transfer code. All of the GPP trajectories in (A) start above 75 Pg C/year at the beginning of the time series in 1850, and all of the NPP trajectories start below 75 Pg C/year. (**B**) Carbon use efficiency, defined as the ratio of NPP to GPP for the same set of models. The multimodel mean is shown with black lines. A scenario in which each year, the relative increase in the NPP flux is reduced by 50% relative to the concurrent relative increase in GPP is shown in purple and identified in the legend as down-regulated (down-reg.) NPP. A damped NPP response of this magnitude is broadly consistent with observations from FACE experiments (*115*).

Considerable evidence suggests that NPP responses to global change drivers are often not proportional to those of GPP. Meta-analysis of free-air carbon dioxide enrichment (FACE) indicates that enhancement of aboveground NPP is, on average, about half the response of light-saturated photosynthesis (*115*). Furthermore, the stem diameter response, a metric closely tied to long-term carbon storage, is approximately half the aboveground NPP response (*115*). Yet, in many models, relative changes in NPP often closely track relative changes in GPP, so that the carbon use efficiency is nearly constant in response to rising atmospheric CO_2_ (Fig. 6). Although variable across sites, several major FACE experiments provide little or no evidence for long-term carbon storage or forest structural changes (*117*, *134*, *135*). Partial decoupling between photosynthesis and carbon accumulation in stem increment has also been observed in forest responses to interannual climate variability (*136*–*138*) and following volcanic eruptions (*139*).

Understanding why some models fail to capture the down-regulation of growth under elevated CO_2_ is crucial for enhancing the realism of models. Allocation schemes that prescribe fixed carbon flows among plant tissues, combined with autotrophic respiration parameterizations that are weakly sensitive to environmental conditions, are likely to produce proportional changes in GPP and NPP. In contrast, more mechanistic treatments of plant respiration (*140*, *141*), explicit representation of nutrient and water limitations on biomass production (*142*), and developmental constraints on plant morphology can allow for greater divergence in GPP and NPP responses to global change drivers.

A longstanding paradigm in land ecosystem models is that plants allocate all available photosynthate to maximize growth (*143*). Although broader ecological frameworks acknowledge that growth and fitness are not equivalent (*144*, *145*), in practice, growth is often treated as a proxy for reproductive success in nearly all of the allocation schemes used in land surface and trait-based plant demography models. This assumption may be reasonable for short-lived annuals, such as grasses and crops, where fitness is closely tied to competitive growth. However, many other environmental pressures likely influence the fitness of long-lived plants like trees and shrubs, which must balance short-term growth with long-term survival. Critical among these episodic but severe events such as droughts, freezing temperatures, windstorms, heatwaves, or lightning strikes—conditions that exert intense selection pressure on plant form and physiology. In such settings, surviving until there is an infrequent but ideal set of years for reproduction may offer greater fitness than maximizing growth, potentially favoring smaller individuals. Over evolutionary timescales, this may have led to the emergence of physiological mechanisms that decouple photosynthesis from growth during transient periods of resource abundance (*116*). These ideas build on existing theory that natural selection in nutrient-poor environments favors plant traits that enhance nutrient use efficiency and limit relative growth rates (*146*). As with animals, where both resource availability and genetic controls influence body size (*147*), genetic constraints on plant development and size are likely significant but remain poorly represented in current models, particularly in describing potential differences in GPP and NPP responses to different global change drivers.

We argue that aligning models with empirical evidence for a dampened NPP and stem growth response under elevated CO_2_ (*115*, *117*, *135*)—illustrated by the purple lines in Fig. 6—would substantially reduce modeled carbon uptake and help reconcile discrepancies with satellite-derived estimates of the net land sink.

This perspective also highlights the importance of size-dependent mortality. At the stand level, faster-growing individuals tend to have shorter life spans (*148*, *149*), although the underlying mechanisms remain uncertain. Environmental stressors may disproportionately affect larger individuals (*150*), and elevated maintenance respiration rates in large trees may compromise their capacity to resist pests or pathogens. Including size-dependent mortality in plant demography models tends to reduce stand-level carbon accumulation compared to models where mortality is age based or random (*149*, *151*–*153*). As Earth system models increasingly integrate plant demographic processes, incorporating this mechanism could further decouple trends in *C*_VEG_ from NPP.

#### Climate-driven disturbance losses

Climate-sensitive disturbances, particularly wildfire, drought, and pest/pathogen outbreaks, have a major impact on forest carbon cycling (*154*–*157*). A recent global study found that stand-replacing disturbance was the largest control on forest biomass turnover rates for 44% of forests and was particularly important in large swaths of temperate and boreal forests (*155*). Current Earth system models struggle to capture disturbance dynamics, likely leading to an underestimate of climate-driven disturbance impacts on carbon stocks (*132*, *133*, *155*, *158*, *159*). Fire is simulated prognostically by only about half of CMIP6 models (*132*, *133*, *160*). In addition, the influence of drought on tree mortality remains a key uncertainty in Earth system model simulations, which often lack a mechanistic representation of the underlying processes, including losses of xylem conductivity and subsequent shifts in plant allocation as trees repair damaged transport tissue (*161*–*163*). To the best of our knowledge, no Earth system model participating in CMIP has simulated insect or pathogen outbreaks. Given the considerable impact of mortality and disturbance on carbon turnover times (*155*) and that the land sink is exceptionally sensitive to turnover time estimates (*164*), one contributing factor to high carbon accumulation rates within land models may be related to substantial “missing” disturbance that is increasing over time in both extent and severity as a consequence of climate change and an expanding human footprint in remote forest areas (*118*–*120*, *159*).

#### Limits on carbon accumulation in soils

Rapid carbon accumulation in litter and soil detrital pools has been proposed in several recent studies as a potential mechanism to reconcile remote sensing and atmospheric constraints on the net land carbon sink (*59*, *60*). Here, we explicitly consider the detrital flux using *S* as defined in Eq. 3 and estimate it to be 60 ± 60% of the flux into living vegetation carbon globally, based on CMIP6 model sink allometry. However, two lines of evidence suggest that this CMIP-derived estimate is likely too high.

First, land surface models overestimate bomb-derived ^14^C in surface soils and underestimate radioactive decay in deeper layers (*165*). This mismatch suggests that excessive carbon is allocated to decadal- and century-scale pools rather than to millennial-scale pools. As a result, models likely overestimate the soil carbon response to historical increases in NPP. Using available radiocarbon and soil carbon stock observations, He *et al.* (*166*) demonstrated that CMIP5 models underestimate the mean age of soil carbon by a factor of over six and overestimate soil carbon sequestration under elevated CO_2_ by about 40%. Reducing uncertainties in the detrital carbon sink will require the next generation of models to explicitly simulate different size classes of woody debris and vertically resolve organic and mineral soil layers. These enhancements will support more robust comparisons with inventory and soil profile observations (*167*), which are essential for better constraining this critical (and highly uncertain) component of the land carbon budget.

Second, most models simulate soil carbon dynamics using first-order kinetics and often omit critical processes that limit carbon uptake (*121*, *168*). Notably, they lack explicit representations of microbial communities and the extracellular enzymes that regulate decomposition of dissolved organic matter (*169*), as well as finite stabilization limits imposed by clay and other mineral surfaces (*122*).

## WAYS TO TEST THE HYPOTHESIS

### Directly measuring long-term changes in ecosystem carbon stocks

There are several ways the weak net land carbon sink hypothesis could be wrong. If new and improved vegetation biomass products show decadal-scale carbon accumulation rates that are about 2 to 2.5 times higher than the remote sensing–derived estimate reported here, then the weak land carbon sink hypothesis would be much less likely. This might be the case if carbon continues accumulating in mature ecosystems even as height and visible and shortwave infrared surface reflectance vegetation indices saturate (and these effects are not well represented in existing biomass retrieval algorithms) or due to inherent uncertainties in using moderate and coarse resolution satellite imagery for change detection (*170*). Alternatively, the vegetation biomass accumulation rates may be robust, but the build-up of detrital pools may be underestimated in inventories and models in the Northern Hemisphere (*60*).

The most direct and efficient way to test the weak land sink hypothesis is to develop new ecosystem carbon stock maps at regional, continental, and global scales designed to track decadal-scale trends. Over the next five years, we anticipate significant breakthroughs in the precision and accuracy of these products, driven by increases in the availability of high-quality satellite observations and advances in machine learning. New lidar (*171*, *172*) and synthetic aperture radar data streams (*173*, *174*) are considerably improving estimates of forest canopy height and structure globally, while an expanding set of visible, near infrared, and shortwave infrared observations from Landsat, Sentinel, and Planet allow for more accurate disturbance mapping and temporal and spatial extrapolation. For this approach to be practical, a concerted international effort is necessary to increase access by scientists to plot-level inventory observations, including precise latitude and longitude coordinate information for sites on public lands (*175*). This information is vital for building more accurate allometry relationships in different forest types, a key source of uncertainty in the current generation of global biomass products. These data are also crucial for reducing uncertainties related to carbon accumulation rates in detrital pools (*60*), for verifying progress toward nationally determined contributions, and for facilitating the reconciliation of country-level forest carbon sink reporting to the UNFCCC with estimates from global models (*176*).

### Independent assessment of the fossil fuel emission inventory

Another path for disproving the weak land sink hypothesis is to demonstrate conclusively that the fossil fuel emission inventory reported in the 2023 Global Carbon Project budget is accurate and does not have a high bias of about 6%. This indirect path is effective because the atmospheric growth rate is measured with high precision, and ocean tracer measurements provide strong constraints on the rates of ocean uptake. Thus, without a lower fossil fuel flux, it becomes challenging to close the global budget with a weak land sink. For this approach to be effective, independent measurements on the extraction, transport, and consumption of gas, oil, and coal must be compiled from companies and public agencies at multiple organizational levels for the top ~20 countries that are the largest fossil fuel emitters. A key component of this verification approach would be the development of an accounting system independent of the methodologies used by government agencies now responsible for self-reporting to the International Energy Agency or the United Nations. Climate TRACE and other organizations are beginning to address this critical need by leveraging remote sensing data and machine learning. However, from the narrower perspective of disproving the weak net land carbon sink hypothesis, this effort is likely to be costlier and logistically complex than an international program designed to map ecosystem carbon stock changes directly. Historically, several countries have resisted increasing access to granular economic and energy data during UNFCCC negotiations due to autonomy and national security concerns (*177*).

Atmospheric and space-based greenhouse gas measurements offer a potentially independent approach for verifying aspects of fossil fuel emission inventories. For methane, advances in aircraft and satellite remote sensing can be transformative due to the importance of point sources and low background methane levels in the free troposphere, which create a relatively high signal-to-noise ratio for emissions targets (*178*, *179*). Considerable advances in space-based CO_2_ monitoring have also been made (*180*, *181*). Still, it is likely to remain challenging for the science community working with the current generation of satellite observations to identify about a 6% global bias in the baseline level of fossil fuel CO_2_ emissions because of limited revisit times, the signal-to-noise ratio of observations, uncertainties in retrieval algorithms, difficulties in parsing biogenic and fossil CO_2_ sources, and the requirement for accurate simulation of regional atmospheric winds needed to connect column mole fraction observations to surface fluxes. In many atmospheric inversion studies, fossil fuel emissions are treated as accurately known in both space and time, with only minor Gaussian uncertainties. However, if fossil fuel emissions have been systematically overestimated by about 6% in recent decades, scenarios with reduced emissions and correspondingly weaker northern land sinks could lead to better alignment between regional inversion-based land sink estimates and bottom-up estimates obtained from inventories, satellites, or land models (*85*). Such scenarios may also improve the agreement between atmospheric simulations and observed intrahemispheric CO_2_ gradients—between industrial centers and remote downwind areas—as well as with multidecadal simulations of the CO_2_ atmospheric growth rate in Earth system models.

## CONCLUSIONS

In the Northern Hemisphere, the more than twofold difference between the atmospheric inversion and remote sensing–derived estimate of the net land carbon sink described here is an important, unresolved puzzle that challenges our fundamental understanding of the global carbon cycle. We provide several lines of evidence that much of this discrepancy can be resolved by a weak net land carbon sink that is distributed mainly in the Northern Hemisphere, together with a relatively small reduction in the magnitude of fossil fuel emissions and a small increase in ocean uptake. This solution weakens the simulated north-south CO_2_ gradient, allowing for a closer agreement between the atmospheric inversion and vegetation carbon–derived sink estimates. A decrease in the fossil fuel flux also generates a global atmospheric O_2_ and CO_2_ budget solution that supports a weak land sink and a more substantial ocean sink. For this budget scenario, the stronger ocean sink inferred from the O_2_ data is closely aligned with a data-constrained ocean model that reproduces chlorofluorocarbon distributions and other tracers reasonably well. The adjustments we propose to other terms to close the global carbon budget with a weak net land sink are within or near the range of current uncertainty estimates for these terms. Although our primary budget analysis focuses on 2000–2019, we speculate that a high bias in fossil fuel emission reporting may have emerged during the second half of the 20th century, coinciding with a period of intensifying international economic competition, the globalization of markets, and expanding trade.

This review also highlights how remote sensing–derived estimates of land cover and biomass carbon are rapidly improving, enabling a new verification of time-integrated fluxes derived from atmospheric measurements and modeling. Over the next 5 years, we anticipate that research in this field will accelerate rapidly, driven by new lidar and synthetic aperture radar satellite missions, high-resolution aircraft and satellite imagery, and advancements in artificial intelligence. With these new data and the availability of high-quality wall-to-wall multispectral reflectance data required for disturbance mapping and extrapolation, we expect that the carbon accumulation rate on land will no longer be the most uncertain term in the global budget. Rather than being inferred as the difference from other terms (*1*), the net land carbon sink will be directly measured. While the location and height of individual trees can already be systematically tracked across North Africa’s dryland biome (*182*), we anticipate that advances in data availability and computing will enable this type of analysis to be extended globally over the next decade.

A strong land carbon sink, as identified in past research, has often been used to support the potential of nature-based climate solutions in meeting climate stabilization targets. However, if the weak land sink hypothesis is correct, then the role of CO_2_ fertilization in enhancing forest carbon stocks might be overestimated. At the same time, projections of carbon accumulation in reforestation and afforestation projects, as incorporated into nationally determined contributions, may also be overly optimistic. A lower-than-expected land carbon sink would also imply that the land biosphere may be closer to transitioning from a net carbon sink to a net source than current models predict. To test the weak land carbon sink hypothesis, we propose accelerating research to measure long-term trends in vegetation and detrital carbon stocks, alongside a coordinated international effort to make plot-level inventory data, including precise location information, publicly accessible for all public lands. Another key implication is the need to revise uncertainty estimates in fossil fuel emission inventories. Reducing these uncertainties will require a dedicated effort to establish more rigorous verification mechanisms for country-level emission reporting.

## Supplementary Materials

### This PDF file includes:

Supplementary Text Fig. S1 Table S1 References
