# Supplementary material for: The weak land carbon sink hypothesis
Source: Sci Adv. 2025 Sep 10;11(37):eadr5489. doi: 10.1126/sciadv.adr5489 (PMC12422194; doi:10.1126/sciadv.adr5489)
Supplement: Supplementary file 1 — Supplementary Text Fig. S1 Table S1 References [file sciadv.adr5489_sm.pdf]

Science Advances
AAAS

Supplementary Materials for

The weak land carbon sink hypothesis

James T. Randerson *et al.*

Corresponding author: James T. Randerson, jranders@uci.edu

Sci. Adv. **10**, eadr5489 (2025)
DOI: 10.1126/sciadv.adr5489

This PDF file includes:

Supplementary Text
Fig. S1
Table S1
References

Supplementary Text

Carbon budget framework

Equation 1 of the 2023 Global Carbon Project budget assessment can be rearranged to express the atmospheric carbon dioxide growth rate (G_{ATM}) as a function of emissions from fossil fuel burning and land use change (E_{FOS} and E_{LUC}), ocean and land carbon sinks (S_{OCEAN} and S_{LAND}), and an imbalance (B_{IM}).

$$G_{ATM} = E_{FOS} + E_{LUC} - S_{OCEAN} - S_{LAND} - B_{IM} \quad \text{(S1)}$$

The imbalance term in Equation S1 arises from the need to quantify uncertainties, given that primarily independent approaches are used to estimate the different budget terms on the right-hand side of the equation. There is a strong historical precedent for separating the net land carbon flux into a component associated with land use change (E_{LUC}) and a residual land sink term (S_{LAND}) associated with the land biosphere's response to changing atmospheric composition and climate (*5*). This separation facilitates compliance with reporting requirements under the United Nations Framework Convention on Climate Change (*183*). It was also used to help close the global carbon budget in IPCC Assessment Reports before the 6th Assessment. In the Global Carbon Project, different models are now employed to estimate the two components of the net land flux. Here, we define the net land sink as the difference of the two terms, with a sign convention that is positive into the land surface:

$$F_{LAND} = S_{LAND} - E_{LUC} \quad \text{(S2)}$$

For the purpose of this review, we focus on net land carbon flux because remote sensing-derived estimates of long-term changes in vegetation carbon stocks likely reflect the influence of a combination of land use change, climate, and atmospheric composition drivers. Also, it is generally easier to extract estimates of the net land flux from atmospheric CO_2 and O_2 inversions and earth system model simulations. In Fig. 1 of the main text, we calculate the time series of F_{LAND} as the difference between the net fossil fuel flux (gross emissions and cement uptake) and accumulation rates in the atmosphere and ocean using estimates of these quantities from the 2023 budget of the Global Carbon Project. For the 2000-2019 period, this yields an estimate of F_{LAND} of 1.6 ± 0.6 Pg C/y. This is similar, but slightly lower, than the estimate obtained by combining Global Carbon Project estimates for S_{LAND} and E_{LUC} that are derived directly from global ecosystem and land use models (1.7 ± 0.9 Pg C/y).

The northern hemisphere land sink derived from a two-box atmosphere model

We used a two-box atmospheric modeling approach described by Ciais et al. (*57*) to extend the northern hemisphere land carbon sink record to 2021. The two-box model is described by the following equation that is derived by assuming the CO_2 growth rate is the same in both hemispheres:

$$F_{LAND,N} = 0.5\left(\left(E_{FOS,N} - E_{FOS,S}\right) + F_{LAND} + \left(S_{OCEAN,S} - S_{OCEAN,N}\right) - \sigma \cdot \frac{1}{\tau} \cdot IG_{ANTH}\right) \quad \text{(S3)}$$

where $F_{LAND,N}$ is the northern hemisphere land carbon sink, $E_{FOS,N}$ and $E_{FOS,S}$ are the fossil fuel emissions in the northern and southern hemispheres, respectively, F_{LAND} is the global net land carbon sink, $S_{OCEAN,N}$ and $S_{OCEAN,S}$ are the net ocean carbon sinks in the northern and southern hemispheres, τ is the interhemispheric atmospheric exchange time, σ is a conversion factor between ppm and Pg C, and IG_{ANTH} is the interhemispheric CO_2 difference caused by human activity (in units of ppm between the northern hemisphere and the southern hemisphere). We used a value of 1.4 y for τ derived from the analysis of SF_6 observations (*12, 57*) and 2.129 Pg C/ppm for σ (*57*).

For $E_{FOS,N}$ and $E_{FOS,S}$, we used the fossil fuel emissions time series from the 2023 Global Carbon Project budget and assigned emissions to either the northern or southern hemisphere based on the location of each country. Bunker fuels, including emissions from aircraft and ships, were distributed

between the two hemispheres in the same proportion as the country-level emissions distribution. To estimate $E_{FOS,N}$ and $E_{FOS,S}$, we also subtracted the cement CO_2 uptake flux in proportion to the hemispheric distribution of emissions. We estimated F_{LAND} as the difference between the fossil fuel flux (after subtracting cement uptake) and the sum of carbon accumulation in the atmosphere and ocean reservoirs using reported values from the Global Carbon Budget.

For the northern and southern hemisphere ocean flux time series, we used estimates derived from the Ocean Circulation Inverse Model (*184*) with optimized parameters used to quantify the spatial pattern and magnitude of the biological pump (*64*). At steady state in 1750, OCIM estimates a northern hemisphere ocean uptake of about 0.41 Pg C/y balanced by a southern hemisphere ocean outgassing of the same magnitude. These natural background fluxes were subtracted from the OCIM time series to isolate the $S_{OCEAN,S} – S_{OCEAN,N}$ gradient associated solely with the anthropogenic perturbation to the ocean carbon flux. We scaled down the magnitude of the OCIM fluxes (by 13%) to match the mean of the Global Carbon Project ocean flux time series during 2000-2019.

To estimate the north-south interhemispheric CO_2 gradient, IG_{OBS}, we used the Mauna Loa and South Pole records from the Scripps Institution of Oceanography CO_2 program (*62, 63*). At each station, we computed annual mean values after filling in missing monthly values using cubic spline interpolation. Using the gap-filled records from each station, we computed the difference between the northern and southern time series as the observed *IG*. The observed *IG* has a natural and an anthropogenic component. In Equation S3, we computed L_N as the anthropogenic perturbation to the land-atmosphere carbon flux. To maintain consistency with all of the other terms in the model (which are also anthropogenic perturbations), it is appropriate to use the anthropogenic component of IG and to estimate this gradient by subtracting the natural component from the observed *IG*: $IG_{ANTH} = IG_{OBS} – IG_{NAT}$.

The natural component of *IG* that operated in a pre-industrial steady state (IG_{NAT}) has multiple contributing processes. These include 1) a chemistry pump in the atmosphere whereby methane, carbon monoxide, and volatile organic compounds emitted from terrestrial ecosystems are oxidized to CO_2 in the remote atmosphere (*185*), 2) a natural ocean carbon cycle associated with interactions between overturning circulation, the biological pump, and air-sea gas exchange (*64*), 3) a land-river-ocean carbon flow by which dissolved inorganic carbon, dissolved organic carbon, particulate organic carbon, and larger organic debris (i.e., dead leaves and wood) are transported to the ocean (*91*) and later decomposed in remote ocean regions, allowing for a steady state interhemispheric atmospheric CO_2 gradient (and land carbon uptake) to balance this flow (*186*), and 4) a biosphere-atmosphere rectifier effect driven by diurnal and annual cycles of terrestrial net ecosystem exchange (*187*). The first three likely contribute to a lower CO_2 concentration in the northern hemisphere than in the remote southern hemisphere, whereas the rectifier effect generates the opposite gradient at surface stations.

We estimated the net contribution of these processes in two different ways and then used the mean value from the two approaches. First, we computed the y-intercept of the fossil fuel - IG_{OBS} relationship from 1959 through 2022 to estimate IG_{NAT}. This yielded a value of -0.4 ± 0.1 ppm (mean intercept and standard error). This result indicates the MLO – SPO difference was negative, with higher southern hemisphere CO_2 concentration for the pre-industrial steady state. A second approach was to take the sum of the component processes from different modeling studies. This yields an estimate of -0.7 ppm (-0.5 ppm from interactions between ocean circulation, chemistry, and biology (*64*), -0.2 from the river-ocean-atmosphere flux loop (*186*), -0.1 from the atmospheric chemical pump (*185*), and +0.1 from the terrestrial biosphere rectifier effect (*23*)). The mean value from the intercept and component modeling approaches was -0.6 ppm after carrying two significant digits on all the terms in the summation. Estimating and removing the influence of IG_{NAT} from IG_{OBS} has the effect of increasing IG_{ANTH} by about 26% during the 1959-2022 period relative to a simulation in which IG_{NAT} is assumed to be zero. Accounting for IG_{NAT} reduces the magnitude of the northern land sink by about same amount. We note that when

computing the y-intercept of the fossil fuel - IG_{OBS} regression, the result is sensitive to the period of the analysis and can vary considerably when different multi-decade periods are used (*12*). We used the full record to minimize this variability, but note that this estimate may be lower than those derived from earlier periods (*12*).

We used a Monte Carlo approach to obtain an uncertainty estimate for $F_{LAND,N}$. In 10^4 simulations with Equation S3, we allowed $E_{FOS,N}$, $E_{FOS,S}$, $S_{OCEAN,N}$, $S_{OCEAN,S}$, and IG_{ANTH} to vary. We assigned Gaussian uncertainties using a Numerical Python random number generator with specified standard deviations derived from estimates reported by the Global Carbon Project for $E_{FOS,N}$ and $E_{FOS,S}$ (5%) and $S_{OCEAN,N}$ and $S_{OCEAN,S}$ (14%). For τ, we assumed an uncertainty estimate of 10%, and for IG_{ANTH}, we assumed uncertainty values of 5% in IG_{OBS} and 25% in IG_{NAT}. We then computed the F_{LAND} for each simulation as the residual difference between fossil fuel emissions and accumulation in the atmosphere and ocean reservoirs. This approach forced the carbon budget to close for each simulation before the computation of $F_{LAND,N}$ in Equation S3. During 1959-2021, this yielded a mean one-sigma uncertainty of ± 31% for $F_{LAND,N}$.

Remote sensing-derived aboveground biomass and net land carbon accumulation

We combined the Jet Propulsion Laboratory (*59*) and Chloris (*49*) biomass products to create a single time series of biomass accumulation at global and hemispheric scales. The JPL product was made publicly available by the authors on Zenodo (https://doi.org/10.5281/zenodo.4161694), and the Chloris product is available upon request from Microsoft's Planetary Computer (https://planetarycomputer.microsoft.com/). The JPL product spans 2000-2019 and includes aboveground (AGB) and belowground (BGB) living biomass pools. Chloris product covers the period from 2003-2019 and represents aboveground biomass. To convert the Chloris dataset to total biomass (C_{VEG}), we used a BGB/AGB ratio of 0.20 for tropical forests, 0.21 for temperate forests, 0.23 for boreal forests, and 0.30 for non-forest ecosystems with an aboveground carbon density of less than 10 Mg C/ha and a tree cover fraction less than 10% (*59*).

To create a single biomass accumulation time series from the two products (ΔC_{VEG}) we used the JPL product from 2000-2003. In 2003, the first year the Chloris data were available, we subtracted or added a constant value from the Chloris time series at each grid cell so that the two products were equal in that first year of temporal overlap. From 2004 through 2019, we used the average of the two records. We assigned a 1-sigma uncertainty estimate of ±70% to the combined product to encompass the individual product means and achieve rough consistency with their reported uncertainty estimates.

To convert ΔC_{VEG} derived from remote sensing to the multi-year (2000-2019) integral of the land carbon sink, ΔC_{LAND}, we multiplied ΔC_{VEG} by a scale factor S (Equation 4 in the main text) of 1.6 ± 0.5. This scale factor was derived from the 17 CMIP6 models listed in Supplementary Table S1. It reflects the internal carbon sink allometry between vegetation and detrital carbon within the models from 2000 to 2019, driven by atmospheric CO_2, climate, and land use changes. To estimate S from each model, we calculated the total global change in vegetation carbon and the global sum of *NBP* from 2000 to 2019 for each model. Defined in this way, S captures carbon changes in forest and non-forest plant functional types. To calculate a multi-model mean estimate, we first calculated the ratio of global *NBP* to ΔC_{VEG} for each model. In a second step, we took the average and standard deviation of the ratios from the set of 17 models. To obtain an uncertainty estimate for ΔC_{LAND}, we combined in a Monte Carlo simulation the uncertainties originating from ΔC_{VEG} and S.

We note that the sum of *NBP* over time is equal to ΔC_{LAND} defined in Equations 1-3. We chose to use the variable *NBP* in our nomenclature in Equation 4 and the model evaluation section of the main text because this variable is more widely reported in CMIP6 models than C_{LITTER} and C_{SOIL}. An alternative approach would have been to compute ΔC_{LAND} using Equation 2 in the main text, based on stock changes for CMIP6 model variables corresponding to vegetation, litter, and soil carbon pools.

Coupled Model Intercomparison Project Phase 6 model analysis

We analyzed Coupled Model Intercomparison Project Phase 6 (CMIP6) earth system models that reported C_{VEG}, C_{LITTER}, C_{SOIL}, and *NBP* variables for historical and SSP 2-4.5 simulations. This set of 17 models is shown in Supplementary Material table S1. To create a continuous time series of Coupled Model Intercomparison Project Phase 6 and *NBP* from 2000-2019, we fused historical and SSP 2-4.5 simulations, using the first ensemble member from each model. For the analysis of carbon use efficiency shown in Fig. 6, we used the subset of these models that also reported GPP and NPP for the idealized 1pctCO2-bgc simulations from C4MIP.

Global land and ocean sinks derived from O_2 and CO_2 trends

To quantify how a reduction in the fossil fuel emissions source would influence the partitioning of land and ocean from dual O_2 and CO_2 atmospheric constraints, we analyzed trends in these trace gases from the Scripps Institution of Oceanography Oxygen Program (*18, 92, 93*). We first extracted all O_2 and CO_2 flask observations for the Alert (ALT), Mauna Loa (MLO), Cape Grim (CGO), and South Pole (SPO) time series from the May 23, 2023 archive. We then fit a cubic spline through these time series and resampled the data to a monthly resolution. We then calculated the annual mean values for the 2000-2019 period. We averaged the four time series to build a representative global time series that captured observed trends in the northern and southern hemispheres.

We used the 2023 Global Carbon Project time series for the fossil fuel vector, computing an annual net fossil fuel flux from the sum of gross fossil fuel emissions and uptake by decomposing cement. We computed a mean annual oxidative ratio (OR) by assuming coal had a value of 1.17, oil a value of 1.44, natural gas a value of 1.95, flaring a value of 1.95, cement a value of 0.0, and other emissions a value of 1.44 (*17, 188*). These factors yielded a mean OR for net fossil fuels that varied between a minimum value of 1.39 in 2013 and a maximum value of 1.42 in 2000. For carbon exchange on land, we assumed the OR was 1.05 (*20, 21, 189*). For ocean outgassing of O_2 due to ocean heat uptake, we used a value of 0.261 ppm/y (46.3 T mol O_2/y) (*188*) and for O_2 production from the reduction of iron and aluminum ores, we used a value of 0.073 ppm/y (16.2 T mol O_2/y) drawing upon annual reports of ore production from the US Geological Survey. We also applied a small adjustment to account for ocean N_2 outgassing and its influence on the O_2/N_2 ratio of the observations. In our analysis, we created a vector for atmospheric observations and fossil fuel emissions spanning the period from the midpoint of 2000 to the midpoint of 2019. We used a ppm to per meg conversion factor of 4.773 and a CO_2 ppm to carbon flux conversion factor of 2.129 Pg C/ppm. We then solved for the length of the land and ocean vectors in O_2 and CO_2 space that closed the budget. To estimate uncertainties for the land and ocean sinks, we performed a Monte Carlo analysis in which we randomly selected different parameters and inputs with the following uncertainty ranges: fossil fuel emissions ± 5%, the sum of outgassing and ore refining ± 25%, fossil fuel emissions oxidative ratio ± 2%, and land carbon flux oxidative ratio ± 2%. The Monte Carlo simulations also cycled over the four different sampling stations described above to capture uncertainties in the observed atmospheric CO_2 and O_2 trends.

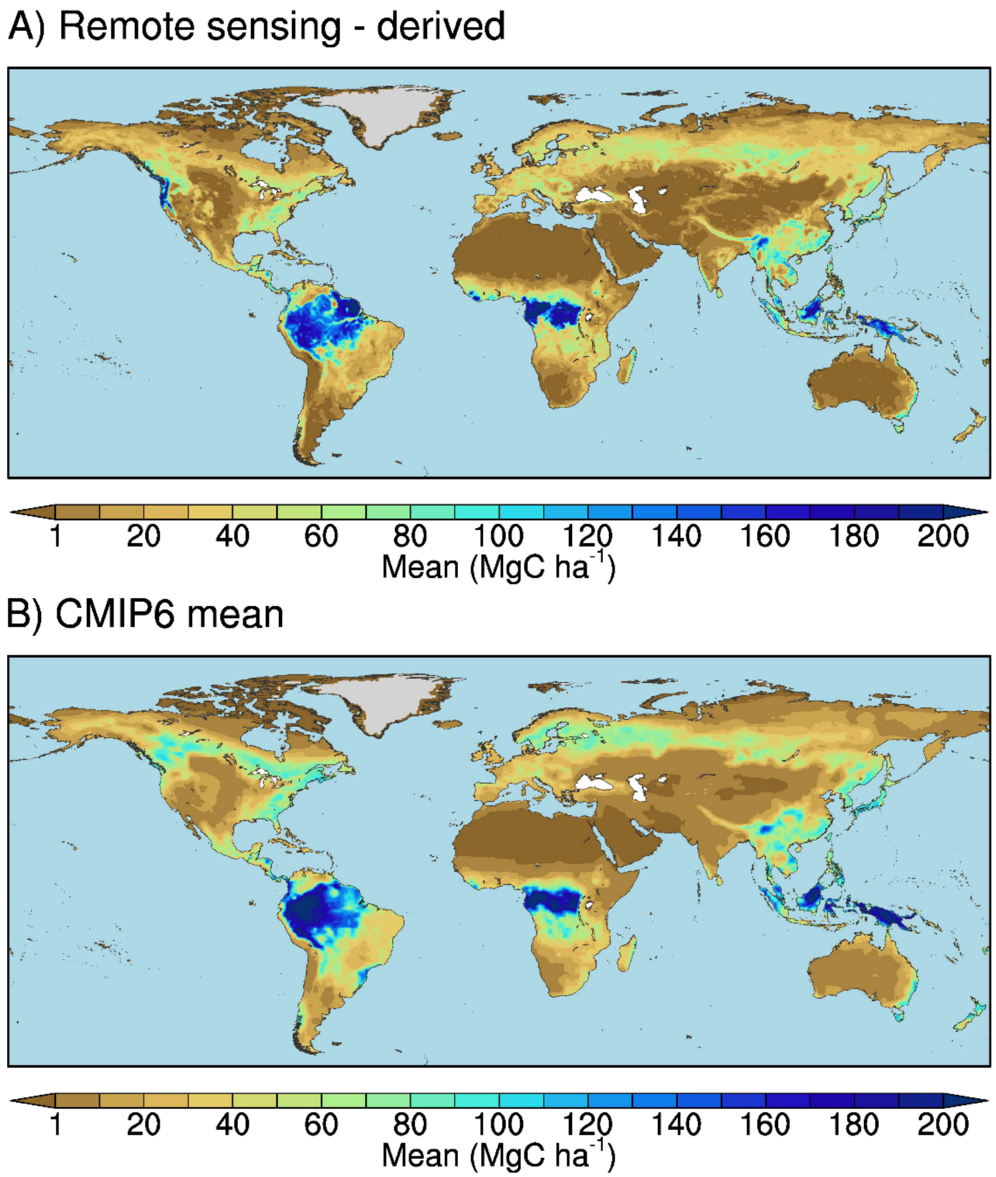

Fig. S1.

Mean vegetation carbon (C_{VEG}) during 2000-2019. Panel A shows a remote sensing-derived estimate created from the average of JPL and Chloris estimates. Panel B shows the multi-model mean of the earth system models from CMIP6 over the same interval.

Model Name	Center	Reference	C_{VEG}	ΣNBP	Spatial correlation (r)
ACCESS-ESM1-5	CSIRO, Australia	Ziehn et al. 2020 (*190*)	12.3	17.5	0.91
BCC-CSM2-MR	BCC, China	Wu et al. 2013 (*191*)	28.0	49.7	0.26
CanESM5	CCCMa, Canada	Swart et al. 2019 (*192*)	25.8	17.0	0.84
CanESM5-1	CCCMa, Canada	Sigmond et al. 2023 (*193*)	25.7	14.4	0.87
CESM2	NCAR, USA	Danabasoglu et al. 2020 (*194*)	10.0	20.2	0.89
CESM2-WACCM	NCAR, USA	Danabasoglu et al. 2020 (*194*)	11.0	18.5	0.90
CMCC-CM2-SR5	CMCC, Italy	Cherchi et al. 2019 (*195*)	9.0	15.0	0.87
CMCC-ESM2	CMCC, Italy	Cherchi et al. 2019 (*195*)	10.4	17.5	0.87
CNRM-ESM2-1	CNRM, CERFACS France	Séférian et al. 2019 (*196*)	27.1	40.1	0.63
EC-Earth3-CC	EC-Earth Consortium, Europe	Döscher et al. 2022 (*197*)	13.9	13.0	0.80
EC-Earth3-Veg	EC-Earth Consortium, Europe	Döscher et al. 2022 (*197*)	11.9	12.7	0.79
IPSL-CM6A-LR	IPSL, France	Boucher et al. 2020 (*198*)	17.9	29.6	0.93
MIROC-ES2L	MIROC, Japan	Hajima et al. 2020 (*199*)	24.8	44.1	0.89
MPI-ESM1-2-LR	MPI-M, Germany	Wieners et al. 2019 (*200*) Mauritsen et al. 2020 (*201*)	11.5	26.4	0.93
NorESM2-LM	NCC, Norway	Seland et al. 2020 (*202*)	11.8	22.4	0.90
NorESM2-MM	NCC, Norway	Seland et al. 2020 (*202*)	8.4	21.2	0.90
UKESM1-0-LL	MOHC, UK	Sellar et al. 2019 (*203*)	8.3	22.6	0.83
	mean		15.7	23.6	0.82
	std. deviation		7.4	11.1	0.16

Table S1.

CMIP6 models reporting C_{VEG} and net biome production (*NBP*) were analyzed here for the net land carbon sink. The final three columns report the global change in C_{VEG} from the middle of 2000 to the middle of 2019 (Pg C), the sum of *NBP* over this same period (Pg C), and the within-model spatial correlation, r, between ΔC_{VEG} and the sum of *NBP* computed using all land grid cells.

REFERENCES AND NOTES

1. W. S. Broecker, T. Takahashi, H. Simpson, T.-H. Peng, Fate of fossil fuel carbon dioxide and the global carbon budget. *Science* **206**, 409–418 (1979).

2. B. Bolin, Changes of land biota and their importance for the carbon cycle: The increase of atmospheric carbon dioxide may partly be due to the expansion of forestry and agriculture. *Science* **196**, 613–615 (1977).

3. G. M. Woodwell, R. Whittaker, W. Reiners, G. E. Likens, C. Delwiche, D. Botkin, The biota and the world carbon budget: The terrestrial biomass appears to be a net source of carbon dioxide for the atmosphere. *Science* **199**, 141–146 (1978).

4. H. Oeschger, U. Siegenthaler, U. Schotterer, A. Gugelmann, A box diffusion model to study the carbon dioxide exchange in nature. *Tellus* **27**, 168–192 (1975).

5. I. C. Prentice, G. Farquhar, M. Fasham, M. L. Goulden, M. Heimann, V. Jaramillo, H. Kheshgi, C. Le Quéré, R. Scholes, D. W. Wallace, "The carbon cycle and atmospheric carbon dioxide" in *Climate Change 2001: The Scientific Basis. Contribution of Working Group I to the Third Assessment Report of the Intergovernmental Panel on Climate Change*, J. Houghton, Y. Ding, D. Griggs, M. Noguer, P. van der Linden, X. Dai, K. Maskell, C. Johnson, Eds. (Cambridge University Press, 2001), chap. 3, pp. 183-237.

6. K. L. Denman, G. Brasseur, A. Chidthaisong, P. Ciais, P. M. Cox, R. E. Dickinson, D. Hauglustaine, C. Heinze, E. Holland, D. Jacob, U. Lohmann, S. Ramachandran, P. L. da Silva Dias, S. C. Wofsy, X. Zhang, "Couplings between changes in the climate system and biogeochemistry" in *Climate Change 2007: The Physical Science Basis. Contribution of Working Group I to the Fourth Assessment Report of the Intergovernmental Panel on Climate Change*, S. Solomon, D. Qin, M. Manning, Z. Chen, M. Marquis, K. B. Averyt, M. Tignor, H. L. Miller, Eds. (Cambridge University Press, 2007), chap. 7, pp. 499-587.

7. P. Ciais, C. Sabine, G. Bala, L. Bopp, V. Brovkin, P. Canadell, A. Chhabra, R. DeFries, J. Galloway, M. Heimann, C. D. Jones, C. Le Quéré, R. Myneni, S. Piao, P. Thornton, "Carbon and other biogeochemical cycles" in *Climate Change 2013: The Physical Science Basis.*

Contribution of Working Group I to the Fifth Assessment Report of the Intergovernmental Panel on Climate Change, T. Stocker, D. Qin, G.-K. Plattner, M. Tignor, S. Allen, J. Boschung, A. Nauels, Y. Xia, V. Bex, P. M. Midgley, Eds. (Cambridge University Press, 2013), chap. 6, pp. 465–570.

8. J. G. Canadell, P. M. S. Monteiro, M. H. Costa, L. Cotrim da Cunha, P. M. Cox, A. V. Eliseev, S. Henson, M. Ishii, S. Jaccard, C. Koven, A. Lohila, P. K. Patra, S. Piao, J. Rogelj, S. Syampungani, S. Zaehle, K. Zickfeld, "Global carbon and other iogeochemical cycles and feedbacks" in *Climate Change 2021: The Physical Science Basis. Contribution of Working Group I to the Sixth Assessment Report of the Intergovernmental Panel on Climate Change*, V. Masson-Delmotte, P. Zhai, A. Pirani, S. I. Connors, C. Péan, S. Berger, N. Caud, Y. Chen, L. Goldfarb, M. I. Gomis, M. Huang, K. Leitzell, E. Lonnoy, J. B. R. Matthews, T. K. Maycock, T. Waterfield, O. Yelekçi, R. Yu, B. Zhou, Eds. (Cambridge, 2021), chap. 6, pp. 673–816.

9. C. Le Quéré, M. R. Raupach, J. G. Canadell, G. Marland, L. Bopp, P. Ciais, T. J. Conway, S. C. Doney, R. A. Feely, P. Foster, Trends in the sources and sinks of carbon dioxide. *Nat. Geosci.* **2**, 831–836 (2009).

10. C. Le Quéré, R. M. Andrew, J. G. Canadell, S. Sitch, J. I. Korsbakken, G. P. Peters, A. C. Manning, T. A. Boden, P. P. Tans, R. A. Houghton, Global carbon budget 2016. *Earth Syst. Sci. Data* **8**, 605–649 (2016).

11. R. J. Francey, C. M. Trudinger, M. Van Der Schoot, R. M. Law, P. B. Krummel, R. L. Langenfelds, L. Paul Steele, C. E. Allison, A. R. Stavert, R. J. Andres, Atmospheric verification of anthropogenic CO_2 emission trends. *Nat. Clim. Chang.* **3**, 520–524 (2013).

12. Y. Wang, M. Li, L. Shen, Accelerating carbon uptake in the Northern Hemisphere: Evidence from the interhemispheric difference of atmospheric CO_2 concentrations. *Tellus B: Chem. Phys. Meteorol.* **65**, 20334 (2013).

13. J. C. Orr, E. Maier-Reimer, U. Mikolajewicz, P. Monfray, J. L. Sarmiento, J. Toggweiler, N. K. Taylor, J. Palmer, N. Gruber, C. L. Sabine, Estimates of anthropogenic carbon uptake from four three-dimensional global ocean models. *Global Biogeochem. Cycles* **15**, 43–60 (2001).

14. J.-C. Dutay, J. L. Bullister, S. C. Doney, J. C. Orr, R. Najjar, K. Caldeira, J.-M. Campin, H. Drange, M. Follows, Y. Gao, Evaluation of ocean model ventilation with CFC-11: Comparison of 13 global ocean models. *Ocean Model.* **4**, 89–120 (2002).

15. N. Gruber, J. L. Sarmiento, T. F. Stocker, An improved method for detecting anthropogenic CO_2 in the oceans. *Global Biogeochem. Cycles* **10**, 809–837 (1996).

16. C. Sabine, R. Key, K. Johnson, F. Millero, A. Poisson, J. L. Sarmiento, D. W. Wallace, C. Winn, Anthropogenic CO_2 inventory of the Indian Ocean. *Global Biogeochem. Cycles* **13**, 179–198 (1999).

17. R. F. Keeling, "Development of an interferometric oxygen analyzer for precise measurement of the atmospheric O_2 mole fraction," thesis, (1988).

18. R. F. Keeling, S. C. Piper, M. Heimann, Global and hemispheric CO_2 sinks deduced from changes in atmospheric O_2 concentration. *Nature* **381**, 218–221 (1996).

19. M. L. Bender, D. T. Ho, M. B. Hendricks, R. Mika, M. O. Battle, P. P. Tans, T. J. Conway, B. Sturtevant, N. Cassar, Atmospheric O_2/N_2 changes, 1993–2002: Implications for the partitioning of fossil fuel CO_2 sequestration. *Global Biogeochem. Cycles* **19**, doi.org/10.1029/2004GB002410 (2005).

20. W. Hockaday, C. Masiello, J. Randerson, R. Smernik, J. Baldock, O. Chadwick, J. Harden, Measurement of soil carbon oxidation state and oxidative ratio by ^{13}C nuclear magnetic resonance. *J. Geophys. Res. Biogeo.* **114**, doi.org/10.1029/2008JG000803 (2009).

21. M. O. Battle, J. W. Munger, M. Conley, E. Sofen, R. Perry, R. Hart, Z. Davis, J. Scheckman, J. Woogerd, K. Graeter, Atmospheric measurements of the terrestrial O_2:CO_2 exchange ratio of a midlatitude forest. *Atmos. Chem. Phys.* **19**, 8687–8701 (2019).

22. P. P. Tans, I. Y. Fung, T. Takahashi, Observational contrains on the global atmospheric CO_2 budget. *Science* **247**, 1431–1438 (1990).

23. K. R. Gurney, R. M. Law, A. S. Denning, P. J. Rayner, D. Baker, P. Bousquet, L. Bruhwiler, Y.-H. Chen, P. Ciais, S. Fan, I. Y. Fung, M. Gloor, M. Heimann, K. Higuchi, J. John, T. Maki, S. Maksyutov, K. Masarie, P. Peylin, M. Prather, B. C. Pak, J. Randerson, J. Sarmiento, S. Taguchi, T. Takahashi, C. W. Yuen, Towards robust regional estimates of CO_2 sources and sinks using atmospheric transport models. *Nature* **415**, 626–630 (2002).

24. B. B. Stephens, K. R. Gurney, P. P. Tans, C. Sweeney, W. Peters, L. Bruhwiler, P. Ciais, M. Ramonet, P. Bousquet, T. Nakazawa, S. Aoki, T. Machida, G. Inoue, N. Vinnichenko, J. Lloyd, A. Jordan, M. Heimann, O. Shibistova, R. L. Langenfelds, L. P. Steele, R. J. Francey, A. S. Denning, Weak northern and strong tropical land carbon uptake from vertical profiles of atmospheric CO_2. *Science* **316**, 1732–1735 (2007).

25. S. Wofsy, M. Goulden, J. Munger, S.-M. Fan, P. Bakwin, B. Daube, S. Bassow, F. Bazzaz, Net exchange of CO_2 in a mid-latitude forest. *Science* **260**, 1314–1317 (1993).

26. R. B. Myneni, C. Keeling, C. J. Tucker, G. Asrar, R. R. Nemani, Increased plant growth in the northern high latitudes from 1981 to 1991. *Nature* **386**, 698–702 (1997).

27. L. Zhou, C. J. Tucker, R. K. Kaufmann, D. Slayback, N. V. Shabanov, R. B. Myneni, Variations in northern vegetation activity inferred from satellite data of vegetation index during 1981 to 1999. *J. Geophys. Res. Atmos.* **106**, 20069–20083 (2001).

28. C. D. Keeling, J. Chin, T. Whorf, Increased activity of northern vegetation inferred from atmospheric CO_2 measurements. *Nature* **382**, 146–149 (1996).

29. D. S. Schimel, Terrestrial ecosystems and the carbon cycle. *Glob. Chang. Biol.* **1**, 77–91 (1995).

30. P. Falkowski, R. Scholes, E. Boyle, J. Canadell, D. Canfield, J. Elser, N. Gruber, K. Hibbard, P. Högberg, S. Linder, The global carbon cycle: A test of our knowledge of Earth as a system. *Science* **290**, 291–296 (2000).

31. R. Bacastow, C. D. Keeling, in *Brookhaven Symposia in Biology* (1973), vol. **24**, pp. 86–135.

32. G. H. Kohlmaier, H. Bröhl, E. O. Siré, M. Plöchl, R. Revelle, Modelling stimulation of plants and ecosystem response to present levels of excess atmospheric CO_2. *Tellus B: Chem. Phys. Meteorol.* **39**, 155–170 (1987).

33. P. Friedlingstein, I. Fung, E. Holland, J. John, G. Brasseur, D. Erickson, D. Schimel, On the contribution of CO_2 fertilization to the missing biospheric sink. *Global Biogeochem. Cycles* **9**, 541–556 (1995).

34. D. Schindler, S. Bayley, The biosphere as an increasing sink for atmospheric carbon: Estimates from increased nitrogen depostion. *Global Biogeochem. Cycles* **7**, 717–733 (1993).

35. A. Townsend, B. Braswell, E. Holland, J. Penner, Spatial and temporal patterns in terrestrial carbon storage due to deposition of fossil fuel nitrogen. *Ecol. Appl.* **6**, 806–814 (1996).

36. E. A. Holland, B. Braswell, J. F. Lamarque, A. Townsend, J. Sulzman, J. F. Müller, F. Dentener, G. Brasseur, H. Levy, J. E. Penner, Variations in the predicted spatial distribution of atmospheric nitrogen deposition and their impact on carbon uptake by terrestrial ecosystems. *J. Geophys. Res. Atmos.* **102**, 15849–15866 (1997).

37. X. Yang, P. E. Thornton, D. M. Ricciuto, F. M. Hoffman, Phosphorus feedbacks constraining tropical ecosystem responses to changes in atmospheric CO_2 and climate. *Geophys. Res. Lett.* **43**, 7205–7214 (2016).

38. L. Gu, D. Baldocchi, S. B. Verma, T. Black, T. Vesala, E. M. Falge, P. R. Dowty, Advantages of diffuse radiation for terrestrial ecosystem productivity. *J. Geophys. Res. Atmos.* **107**, ACL 2-1–ACL 2-23 (2002).

39. G. D. Farquhar, M. L. Roderick, Pinatubo, diffuse light, and the carbon cycle. *Science* **299**, 1997–1998 (2003).

40. L. M. Mercado, N. Bellouin, S. Sitch, O. Boucher, C. Huntingford, M. Wild, P. M. Cox, Impact of changes in diffuse radiation on the global land carbon sink. *Nature* **458**, 1014–1017 (2009).

41. S. Piao, P. Friedlingstein, P. Ciais, N. Viovy, J. Demarty, Growing season extension and its impact on terrestrial carbon cycle in the Northern Hemisphere over the past 2 decades. *Global Biogeochem. Cycles* **21**, doi.org/10.1029/2006GB002888 (2007).

42. A. Gonsamo, J. M. Chen, Y. W. Ooi, Peak season plant activity shift towards spring is reflected by increasing carbon uptake by extratropical ecosystems. *Glob. Chang. Biol.* **24**, 2117–2128 (2018).

43. S. Sitch, P. Cox, W. Collins, C. Huntingford, Indirect radiative forcing of climate change through ozone effects on the land-carbon sink. *Nature* **448**, 791–794 (2007).

44. R. A. Houghton, The worldwide extent of land-use change. *Bioscience* **44**, 305–313 (1994).

45. R. A. Houghton, J. I. House, J. Pongratz, G. R. Van Der Werf, R. S. Defries, M. C. Hansen, C. Le Quéré, N. Ramankutty, Carbon emissions from land use and land-cover change. *Biogeosciences* **9**, 5125–5142 (2012).

46. D. Huntzinger, A. Michalak, C. Schwalm, P. Ciais, A. King, Y. Fang, K. Schaefer, Y. Wei, R. Cook, J. Fisher, Uncertainty in the response of terrestrial carbon sink to environmental drivers undermines carbon-climate feedback predictions. *Sci. Rep.* **7**, 4765 (2017).

47. S. Ruehr, T. F. Keenan, C. Williams, Y. Zhou, X. Lu, A. Bastos, J. G. Canadell, I. C. Prentice, S. Sitch, C. Terrer, Evidence and attribution of the enhanced land carbon sink. *Nat. Rev. Earth Environ.* **4**, 518–534 (2023).

48. A. P. Walker, M. G. De Kauwe, A. Bastos, S. Belmecheri, K. Georgiou, R. F. Keeling, S. M. McMahon, B. E. Medlyn, D. J. Moore, R. J. Norby, Integrating the evidence for a terrestrial carbon sink caused by increasing atmospheric CO_2. *New Phytol.* **229**, 2413–2445 (2021).

49. A. Baccini, W. Walker, L. Carvalho, M. Farina, D. Sulla-Menashe, R. Houghton, Tropical forests are a net carbon source based on aboveground measurements of gain and loss. *Science* **358**, 230–234 (2017).

50. L. V. Gatti, C. L. Cunha, L. Marani, H. L. Cassol, C. G. Messias, E. Arai, A. S. Denning, L. S. Soler, C. Almeida, A. Setzer, L. G. Domingues, L. S. Basso, J. B. Miller, M. Gloor, C. S. C. Correia, G. Tejada, R. A. L. Neves, R. Rajao, F. Nunes, B. S. S. Filho, J. Schmitt, C. Nobre, S. M. Corrêa, A. H. Sanches, L. E. O. C. Aragão, L. Anderson, C. von Randow, S. P. Crispim, F. M. Silva, G. B. M. Machado, Increased Amazon carbon emissions mainly from decline in law enforcement. *Nature* **621**, 318–323 (2023).

51. G. Hurtt, S. W. Pacala, P. R. Moorcroft, J. Caspersen, E. Shevliakova, R. Houghton, B. Moore III, Projecting the future of the US carbon sink. *Proc. Natl. Acad. Sci. U.S.A.* **99**, 1389–1394 (2002).

52. J. P. Caspersen, S. W. Pacala, J. C. Jenkins, G. C. Hurtt, P. R. Moorcroft, R. A. Birdsey, Contributions of land-use history to carbon accumulation in U.S. forests. *Science* **290**, 1148–1151 (2000).

53. Y. Li, S. Piao, L. Z. Li, A. Chen, X. Wang, P. Ciais, L. Huang, X. Lian, S. Peng, Z. Zeng, Divergent hydrological response to large-scale afforestation and vegetation greening in China. *Sci. Adv.* **4**, eaar4182 (2018).

54. F. Lu, H. Hu, W. Sun, J. Zhu, G. Liu, W. Zhou, Q. Zhang, P. Shi, X. Liu, X. Wu, Effects of national ecological restoration projects on carbon sequestration in China from 2001 to 2010. *Proc. Natl. Acad. Sci. U.S.A.* **115**, 4039–4044 (2018).

55. X. Zhang, M. Brandt, Y. Yue, X. Tong, K. Wang, R. Fensholt, The carbon sink potential of southern China after two decades of afforestation. *Earth's Futures* **10**, e2022EF002674 (2022).

56. P. Friedlingstein, M. O'sullivan, M. W. Jones, R. M. Andrew, D. C. Bakker, J. Hauck, P. Landschützer, C. Le Quéré, I. T. Luijkx, G. P. Peters, Global carbon budget 2023. *Earth Syst.Sci. Data* **15**, 5301–5369 (2023).

57. P. Ciais, J. Tan, X. Wang, C. Roedenbeck, F. Chevallier, S.-L. Piao, R. Moriarty, G. Broquet, C. Le Quéré, J. Canadell, Five decades of northern land carbon uptake revealed by the interhemispheric CO_2 gradient. *Nature* **568**, 221–225 (2019).

58. B. Poulter, G. Murray-Tortarolo, D. J. Hayes, P. Ciais, R. M. Andrew, A. Bastos, B. Byrne, D. Butman, J. G. Canadell, A. Chatterjee, The North American Greenhouse Gas Budget: Emissions, removals, and integration for CO_2, CH_4, and N_2O (2010–2019): Results from the second REgional carbon cycle assessment and processes study (RECCAP2). *Global Biogeochem. Cycles* **39**, e2024GB008310 (2025).

59. L. Xu, S. S. Saatchi, Y. Yang, Y. Yu, J. Pongratz, A. A. Bloom, K. Bowman, J. Worden, J. Liu, Y. Yin, Changes in global terrestrial live biomass over the 21st century. *Sci. Adv.* **7**, eabe9829 (2021).

60. Y. M. Bar-On, X. Li, M. O'Sullivan, J.-P. Wigneron, S. Sitch, P. Ciais, C. Frankenberg, W. W. Fischer, Recent gains in global terrestrial carbon stocks are mostly stored in nonliving pools. *Science* **387**, 1291–1295 (2025).

61. C. D. Jones, V. Arora, P. Friedlingstein, L. Bopp, V. Brovkin, J. Dunne, H. Graven, F. Hoffman, T. Ilyina, J. G. John, C4MIP–The coupled climate–carbon cycle model intercomparison project: Experimental protocol for CMIP6. *Geosci. Model Dev.* **9**, 2853–2880 (2016).

62. C. D. Keeling, S. C. Piper, R. B. Bacastow, M. Wahlen, T. P. Whorf, M. Heimann, H. A. Meijer, "Exchanges of atmospheric CO_2 and $^{13}CO_2$ with the terrestrial biosphere and oceans from 1978 to 2000. I. Global aspects" (Scripps Institution of Oceanography, 2001).

63. C. D. Keeling, S. C. Piper, R. B. Bacastow, M. Wahlen, T. P. Whorf, M. Heimann, H. A. Meijer, "Atmospheric CO_2 and $^{13}CO_2$ exchange with the terrestrial biosphere and oceans from 1978 to 2000: Observations and carbon cycle implications" in *A History of Atmospheric CO_2 and Its Effects on Plants, Animals, and Ecosystems*, I. T. Baldwin, E.-D. Schulze, M. M. Caldwell, G. Heldmaier, R. B. Jackson, O. L. Lange, H. A. Mooney, U. Sommer, J. R. Ehleringer, M. D. Dearing, T. E. Cerling, Eds. (Springer, 2005), pp. 83–113.

64. W. Wang, W. Fu, A. Frédéric, L. Moigne, R. Letscher, Y. Liu, J. Tang, F. Primeau, Biological carbon pump estimate based on multidecadal hydrographic data. *Nature* **624**, 579–585 (2023).

65. H. Yang, P. Ciais, F. Frappart, X. Li, M. Brandt, R. Fensholt, L. Fan, S. Saatchi, S. Besnard, Z. Deng, Global increase in biomass carbon stock dominated by growth of northern young forests over past decade. *Nat. Geosci.* **16**, 886–892 (2023).

66. E. A. Schuur, B. W. Abbott, R. Commane, J. Ernakovich, E. Euskirchen, G. Hugelius, G. Grosse, M. Jones, C. Koven, V. Leshyk, Permafrost and climate change: Carbon cycle feedbacks from the warming Arctic. *Annu. Rev. Env. Resour.* **47**, 343–371 (2022).

67. Y. Pan, R. A. Birdsey, O. L. Phillips, R. A. Houghton, J. Fang, P. E. Kauppi, H. Keith, W. A. Kurz, A. Ito, S. L. Lewis, The enduring world forest carbon sink. *Nature* **631**, 563–569 (2024).

68. W. Fu, J. K. Moore, F. Primeau, N. Collier, O. O. Ogunro, F. M. Hoffman, J. T. Randerson, Evaluation of ocean biogeochemistry and carbon cycling in CMIP earth system models with the International Ocean Model Benchmarking (IOMB) software system. *J. Geophys. Res. Oceans* **127**, e2022JC018965 (2022).

69. N. Gruber, D. Clement, B. R. Carter, R. A. Feely, S. Van Heuven, M. Hoppema, M. Ishii, R. M. Key, A. Kozyr, S. K. Lauvset, The oceanic sink for anthropogenic CO_2 from 1994 to 2007. *Science* **363**, 1193–1199 (2019).

70. J. Terhaar, T. L. Frölicher, F. Joos, Observation-constrained estimates of the global ocean carbon sink from Earth system models. *Biogeosciences* **19**, 4431–4457 (2022).

71. R. J. Andres, T. A. Boden, F.-M. Bréon, P. Ciais, S. Davis, D. Erickson, J. S. Gregg, A. Jacobson, G. Marland, J. Miller, A synthesis of carbon dioxide emissions from fossil-fuel combustion. *Biogeosciences* **9**, 1845–1871 (2012).

72. C. G. Gaddy, B. W. Ickes, Russia's virtual economy. *Foreign Aff.* **77**, 53 (1998).

73. T. G. Rawski, What is happening to China's GDP statistics? *China Econ. Rev.* **12**, 347–354 (2001).

74. Z. Li, Re-estimating the growth rate of the Chinese economy from a provincial perspective by correcting two biases. *Ann. Econ. Financ.* **25**, 327–344 (2024).

75. A. A. Coremberg, Argentina was not the productivity and economic growth champion of Latin America. *Int. Prod. Monit.* **33**, 77–90 (2017).

76. G. Cai, X. Li, B. Lin, D. Luo, GDP manipulation, political incentives, and earnings management. *J. Account. Public Policy* **41**, 106949 (2022).

77. M. Jerven, *Poor Numbers: How We Are Misled by African Development Statistics and What to Do About It* (Cornell University Press, 2013).

78. Y. Stiller, Bargaining power in a globalized world: The effect of global value chains in trade negotiations. *Business and Politics* **25**, 173–194 (2023).

79. L. R. Martinez, How much should we trust the dictator's GDP growth estimates? *J. Pol. Econ.* **130**, 2731–2769 (2022).

80. J. Kraft, A. Kraft, On the relationship between energy and GNP. *J. Energy Dev.* **3**, 401–403 (1978).

81. H. Allcott, A. Collard-Wexler, S. D. O'Connell, How do electricity shortages affect industry? Evidence from India *Am. Econ. Rev.* **106**, 587–624 (2016).

82. M. R. Raupach, G. Marland, P. Ciais, C. Le Quéré, J. G. Canadell, G. Klepper, C. B. Field, Global and regional drivers of accelerating CO_2 emissions. *Proc. Natl. Acad. Sci. U.S.A.* **104**, 10288–10293 (2007).

83. D. Guan, Z. Liu, Y. Geng, S. Lindner, K. Hubacek, The gigatonne gap in China's carbon dioxide inventories. *Nat. Clim. Chang.* **2**, 672–675 (2012).

84. R. M. Andrew, A comparison of estimates of global carbon dioxide emissions from fossil carbon sources. *Earth Syst. Sci. Data* **12**, 1437–1465 (2020).

85. T. Saeki, P. K. Patra, Implications of overestimated anthropogenic CO_2 emissions on East Asian and global land CO_2 flux inversion. *Geosci. Lett.* **4**, 9 (2017).

86. P. K. Patra, T. Saeki, E. J. Dlugokencky, K. Ishijima, T. Umezawa, A. Ito, S. Aoki, S. Morimoto, E. A. Kort, A. Crotwell, Regional methane emission estimation based on observed atmospheric concentrations (2002-2012). *JMSJ Ser.II* **94**, 91–113 (2016).

87. O. B. Toon, C. G. Bardeen, A. Robock, L. Xia, H. Kristensen, M. McKinzie, R. Peterson, C. S. Harrison, N. S. Lovenduski, R. P. Turco, Rapidly expanding nuclear arsenals in Pakistan and India portend regional and global catastrophe. *Sci. Adv.* **5**, eaay5478 (2019).

88. G. Churkina, A. Organschi, C. P. Reyer, A. Ruff, K. Vinke, Z. Liu, B. K. Reck, T. Graedel, H. J. Schellnhuber, Buildings as a global carbon sink. *Nat. Sustain.* **3**, 269–276 (2020).

89. D. J. Nowak, D. E. Crane, Carbon storage and sequestration by urban trees in the USA. *Environ. Pollut.* **116**, 381–389 (2002).

90. V. Vasenev, Y. Kuzyakov, Urban soils as hot spots of anthropogenic carbon accumulation: Review of stocks, mechanisms and driving factors. *Land Degrad. Dev.* **29**, 1607–1622 (2018).

91. P. Regnier, L. Resplandy, R. G. Najjar, P. Ciais, The land-to-ocean loops of the global carbon cycle. *Nature* **603**, 401–410 (2022).

92. R. F. Keeling, S. J. Walker, B. Paplawsky, "Span Sensitivity of the Scripps Interferometric Oxygen Analyzer" (https://escholarship.org/uc/item/7tt993fj, Scripps Institution of Oceanography, 2020).

93. R. F. Keeling, A. C. Manning, W. J. Paplawsky, A. C. Cox, On the long-term stability of reference gases for atmospheric O_2/N_2 and CO_2 measurements. *Tellus B: Chem. Phys. Meteorol.* **59**, 3–14 (2007).

94. S. B. Verma, D. D. Baldocchi, D. E. Anderson, D. R. Matt, R. J. Clement, Eddy fluxes of CO_2, water vapor, and sensible heat over a deciduous forest. *Bound.-Lay. Meteorol.* **36**, 71–91 (1986).

95. D. Baldocchi, E. Falge, L. Gu, R. Olson, D. Hollinger, S. Running, P. Anthoni, C. Bernhofer, K. Davis, R. Evans, FLUXNET: A new tool to study the temporal and spatial variability of

ecosystem-scale carbon dioxide, water vapor, and energy flux densities. *Bull. Am. Meteorol. Soc.* **82**, 2415–2434 (2001).

96. M. L. Goulden, J. W. Munger, S. M. Fan, B. C. Daube, S. C. Wofsy, Measurements of carbon sequestration by long-term eddy covariance: Methods and a critical evaluation of accuracy. *Glob. Chang. Biol.* **2**, 169–182 (1996).

97. M. L. Goulden, S. D. Miller, H. R. Da Rocha, Nocturnal cold air drainage and pooling in a tropical forest. *J. Geophys. Res. Atmos.* **111**, D08S04 (2006).

98. M. Aubinet, Eddy covariance CO2 flux measurements in nocturnal conditions: An analysis of the problem. *Ecol. Appl.* **18**, 1368–1378 (2008).

99. J. Jian, V. Bailey, K. Dorheim, A. G. Konings, D. Hao, A. N. Shiklomanov, A. Snyder, M. Steele, M. Teramoto, R. Vargas, B.-L. Ben, Historically inconsistent productivity and respiration fluxes in the global terrestrial carbon cycle. *Nat. Commun.* **13**, 1733 (2022).

100. M. Jung, M. Reichstein, H. A. Margolis, A. Cescatti, A. D. Richardson, M. A. Arain, A. Arneth, C. Bernhofer, D. Bonal, J. Chen, Global patterns of land-atmosphere fluxes of carbon dioxide, latent heat, and sensible heat derived from eddy covariance, satellite, and meteorological observations. *J. Geophys. Res. Biogeosci.* **116**, doi.org/10.1029/2010JG001566 (2011).

101. M. Jung, C. Schwalm, M. Migliavacca, S. Walther, G. Camps-Valls, S. Koirala, P. Anthoni, S. Besnard, P. Bodesheim, N. Carvalhais, F. Chevallier, F. Gans, D. S. Goll, V. Haverd, P. Köhler, K. Ichii, A. K. Jain, J. Liu, D. Lombardozzi, J. E. Nabel, J. A. Nelson, M. O'Sullivan, M. Pallandt, D. Papale, W. Peters, J. Pongratz, C. Rödenbeck, S. Sitch, G. Tramontana, A. Walker, U. Weber, M. Reichstein, Scaling carbon fluxes from eddy covariance sites to globe: Synthesis and evaluation of the FLUXCOM approach. *Biogeosciences* **17**, 1343–1365 (2020).

102. L. B. Hutley, J. Beringer, S. Fatichi, S. J. Schymanski, M. Northwood, Gross primary productivity and water use efficiency are increasing in a high rainfall tropical savanna. *Glob. Chang. Biol.* **28**, 2360–2380 (2022).

103. C. Chen, W. J. Riley, I. C. Prentice, T. F. Keenan, CO_2 fertilization of terrestrial photosynthesis inferred from site to global scales. *Proc. Natl. Acad. Sci.* **119**, e2115627119 (2022).

104. Z. Zhu, S. Piao, R. B. Myneni, M. Huang, Z. Zeng, J. G. Canadell, P. Ciais, S. Sitch, P. Friedlingstein, A. Arneth, Greening of the Earth and its drivers. *Nat. Clim. Chang.* **6**, 791–795 (2016).

105. S. Piao, X. Wang, T. Park, C. Chen, X. Lian, Y. He, J. W. Bjerke, A. Chen, P. Ciais, H. Tømmervik, Characteristics, drivers and feedbacks of global greening. *Nat. Rev. Earth Environ.* **1**, 14–27 (2020).

106. S. Cao, M. Li, Z. Zhu, Z. Wang, J. Zha, W. Zhao, Z. Duanmu, J. Chen, Y. Zheng, Y. Chen, Spatiotemporally consistent global dataset of the GIMMS leaf area index (GIMMS LAI4g) from 1982 to 2020. *Earth Syst. Sci. Data* **15**, 4877–4899 (2023).

107. L. Cheng, L. Zhang, Y.-P. Wang, J. G. Canadell, F. H. Chiew, J. Beringer, L. Li, D. G. Miralles, S. Piao, Y. Zhang, Recent increases in terrestrial carbon uptake at little cost to the water cycle. *Nat. Commun.* **8**, 110 (2017).

108. K. A. Endsley, M. Zhao, J. S. Kimball, S. Devadiga, Continuity of global MODIS terrestrial primary productivity estimates in the VIIRS era using model-data fusion. *J. Geophys. Res. G: Biogeosciences.* **128**, e2023JG007457 (2023).

109. Z. Wang, J. Peñuelas, T. Tagesson, W. Smith, M. Wu, W. He, S. Sitch, S. Wang, Evolution of global terrestrial gross primary productivity trend. *Ecosyst. Health Sustain.* **10**, 0278 (2024).

110. J. Campbell, J. Berry, U. Seibt, S. J. Smith, S. Montzka, T. Launois, S. Belviso, L. Bopp, M. Laine, Large historical growth in global terrestrial gross primary production. *Nature* **544**, 84–87 (2017).

111. J. Randerson, C. Field, I. Fung, P. Tans, Increases in early season ecosystem uptake explain recent changes in the seasonal cycle of atmospheric CO_2 at high northern latitudes. *Geophys. Res. Lett.* **26**, 2765–2768 (1999).

112. H. Graven, R. Keeling, S. Piper, P. Patra, B. Stephens, S. Wofsy, L. Welp, C. Sweeney, P. Tans, J. Kelley, B. C. Daube, E. A. Kort, G. W. Santoni, J. D. Bent, Enhanced seasonal exchange of CO_2 by northern ecosystems since 1960. *Science* **341**, 1085–1089 (2013).

113. M. Forkel, N. Carvalhais, C. Rödenbeck, R. Keeling, M. Heimann, K. Thonicke, S. Zaehle, M. Reichstein, Enhanced seasonal CO_2 exchange caused by amplified plant productivity in northern ecosystems. *Science* **351**, 696–699 (2016).

114. L. He, B. Byrne, Y. Yin, J. Liu, C. Frankenberg, Remote-sensing derived trends in gross primary production explain increases in the CO_2 seasonal cycle amplitude. *Global Biogeochem. Cycles* **36**, e2021GB007220 (2022).

115. E. A. Ainsworth, A. Rogers, The response of photosynthesis and stomatal conductance to rising [CO_2]: Mechanisms and environmental interactions. *Plant Cell Environ.* **30**, 258–270 (2007).

116. C. Körner, Paradigm shift in plant growth control. *Curr. Opin. Plant Biol.* **25**, 107–114 (2015).

117. M. Jiang, B. E. Medlyn, J. E. Drake, R. A. Duursma, I. C. Anderson, C. V. Barton, M. M. Boer, Y. Carrillo, L. Castañeda-Gómez, L. Collins, K. Y. Crous, M. G. de Kauwe, B. dos Santos, K. M. Emmerson, S. L. Facey, A. N. Gherlenda, T. E. Gimeno, S. Hasegawa, S. N. Johnson, A. Kännaste, C. A. Macdonald, K. Mahmud, B. D. Moore, L. Nazaries, E. H. J. Neilson, U. N. Nielsen, Ü. Niinemets, N. J. Noh, R. Ochoa-Hueso, V. S. Pathare, E. Pendall, J. Pihlblad, J. Piñeiro, J. R. Powell, S. A. Power, P. B. Reich, A. A. Renchon, M. Riegler, R. Rinnan, P. D. Rymer, R. L. Salomón, B. K. Singh, B. Smith, M. G. Tjoelker, J. K. M. Walker, A. Wujeska-Klause, J. Yang, S. Zaehle, D. S. Ellsworth, The fate of carbon in a mature forest under carbon dioxide enrichment. *Nature* **580**, 227–231 (2020).

118. M. Patacca, M. Lindner, M. E. Lucas-Borja, T. Cordonnier, G. Fidej, B. Gardiner, Y. Hauf, G. Jasinevičius, S. Labonne, E. Linkevičius, Significant increase in natural disturbance impacts on European forests since 1950. *Glob. Chang. Biol.* **29**, 1359–1376 (2023).

119. V. Iglesias, J. K. Balch, W. R. Travis, US fires became larger, more frequent, and more widespread in the 2000s. *Sci. Adv.* **8**, eabc0020 (2022).

120. M. W. Jones, S. Veraverbeke, N. Andela, S. H. Doerr, C. Kolden, G. Mataveli, M. L. Pettinari, C. Le Quéré, T. M. Rosan, G. R. van der Werf, Global rise in forest fire emissions linked to climate change in the extratropics. *Science* **386**, eadl5889 (2024).

121. W. R. Wieder, S. D. Allison, E. A. Davidson, K. Georgiou, O. Hararuk, Y. He, F. Hopkins, Y. Luo, M. J. Smith, B. Sulman, Explicitly representing soil microbial processes in Earth system models. *Global Biogeochem. Cycles* **29**, 1782–1800 (2015).

122. K. Georgiou, R. B. Jackson, O. Vindušková, R. Z. Abramoff, A. Ahlström, W. Feng, J. W. Harden, A. F. Pellegrini, H. W. Polley, J. L. Soong, Global stocks and capacity of mineral-associated soil organic carbon. *Nat. Commun.* **13**, 3797 (2022).

123. J. E. Kim, J. A. Wang, Y. Li, C. I. Czimczik, J. T. Randerson, Wildfire-induced increases in photosynthesis in boreal forest ecosystems of North America. *Glob. Chang. Biol.* **30**, e17151 (2024).

124. S. Zimov, S. Davidov, G. Zimova, A. Davidova, F. Chapin III, M. Chapin, J. Reynolds, Contribution of disturbance to increasing seasonal amplitude of atmospheric CO_2. *Science* **284**, 1973–1976 (1999).

125. L. Welp, J. Randerson, H. Liu, The sensitivity of carbon fluxes to spring warming and summer drought depends on plant functional type in boreal forest ecosystems. *Agric. For. Meteorol.* **147**, 172–185 (2007).

126. X. J. Walker, J. L. Baltzer, S. G. Cumming, N. J. Day, C. Ebert, S. Goetz, J. F. Johnstone, S. Potter, B. M. Rogers, E. A. Schuur, M. R. Turetsky, M. C. Mack, Increasing wildfires threaten historic carbon sink of boreal forest soils. *Nature* **572**, 520–523 (2019).

127. J. M. Gray, S. Frolking, E. A. Kort, D. K. Ray, C. J. Kucharik, N. Ramankutty, M. A. Friedl, Direct human influence on atmospheric CO_2 seasonality from increased cropland productivity. *Nature* **515**, 398–401 (2014).

128. N. Zeng, F. Zhao, G. J. Collatz, E. Kalnay, R. J. Salawitch, T. O. West, L. Guanter, Agricultural green revolution as a driver of increasing atmospheric CO_2 seasonal amplitude. *Nature* **515**, 394–397 (2014).

129. J. D. Müller, N. Gruber, B. Carter, R. Feely, M. Ishii, N. Lange, S. K. Lauvset, A. Murata, A. Olsen, F. F. Pérez, Decadal trends in the oceanic storage of anthropogenic carbon from 1994 to 2014. *AGU Adv.* **4**, e2023AV000875 (2023).

130. S. Sitch, M. O'sullivan, E. Robertson, P. Friedlingstein, C. Albergel, P. Anthoni, A. Arneth, V. K. Arora, A. Bastos, V. Bastrikov, Trends and drivers of terrestrial sources and sinks of carbon dioxide: An overview of the TRENDY project. *Global Biogeochem. Cycles* **38**, e2024GB008102 (2024).

131. W. Kolby Smith, S. C. Reed, C. C. Cleveland, A. P. Ballantyne, W. R. Anderegg, W. R. Wieder, Y. Y. Liu, S. W. Running, Large divergence of satellite and Earth system model estimates of global terrestrial CO_2 fertilization. *Nat. Clim. Chang.* **6**, 306–310 (2016).

132. J. A. Wang, A. Baccini, M. Farina, J. T. Randerson, M. A. Friedl, Disturbance suppresses the aboveground carbon sink in North American boreal forests. *Nat. Clim. Chang.* **11**, 435–441 (2021).

133. C. Wu, S. R. Coffield, M. L. Goulden, J. T. Randerson, A. T. Trugman, W. R. Anderegg, Uncertainty in US forest carbon storage potential due to climate risks. *Nat. Geosci.* **16**, 422–429 (2023).

134. C. Korner, R. Asshoff, O. Bignucolo, S. Hättenschwiler, S. G. Keel, S. Peláez-Riedl, S. Pepin, R. T. Siegwolf, G. Zotz, Carbon flux and growth in mature deciduous forest trees exposed to elevated CO_2. *Science* **309**, 1360–1362 (2005).

135. R. J. Norby, J. M. Warren, C. M. Iversen, J. Childs, S. S. Jawdy, A. P. Walker, Forest stand and canopy development unaltered by 12 years of CO_2 enrichment. *Tree Physiol.* **42**, 428–440 (2022).

136. A. V. Rocha, M. L. Goulden, A. L. Dunn, S. C. Wofsy, On linking interannual tree ring variability with observations of whole-forest CO_2 flux. *Glob. Chang. Biol.* **12**, 1378–1389 (2006).

137. F. Babst, O. Bouriaud, D. Papale, B. Gielen, I. A. Janssens, E. Nikinmaa, A. Ibrom, J. Wu, C. Bernhofer, B. Köstner, T. Grünwald, G. Seufert, P. Ciais, D. Frank, Above-ground woody carbon sequestration measured from tree rings is coherent with net ecosystem productivity at five eddy-covariance sites. *New Phytol.* **201**, 1289–1303 (2014).

138. A. Cabon, S. A. Kannenberg, A. Arain, F. Babst, D. Baldocchi, S. Belmecheri, N. Delpierre, R. Guerrieri, J. T. Maxwell, S. McKenzie, F. C. Meinzer, D. J. P. Moore, C. Pappas, A. V. Rocha, P. Szejner, M. Ueyama, D. Ulrich, C. Vincke, S. L. Voelker, J. Wei, D. Woodruff, W. R. L. Anderegg, Cross-biome synthesis of source versus sink limits to tree growth. *Science* **376**, 758–761 (2022).

139. A. Cabon, W. R. Anderegg, Large volcanic eruptions elucidate physiological controls of tree growth and photosynthesis. *Ecol. Lett.* **26**, 257–267 (2023).

140. N. G. Smith, J. S. Dukes, Plant respiration and photosynthesis in global-scale models: Incorporating acclimation to temperature and CO_2. *Glob. Chang. Biol.* **19**, 45–63 (2013).

141. O. K. Atkin, K. J. Bloomfield, P. B. Reich, M. G. Tjoelker, G. P. Asner, D. Bonal, G. Bönisch, M. G. Bradford, L. A. Cernusak, E. G. Cosio, Global variability in leaf respiration in relation to climate, plant functional types and leaf traits. *New Phytol.* **206**, 614–636 (2015).

142. S. Zaehle, B. E. Medlyn, M. G. De Kauwe, A. P. Walker, M. C. Dietze, T. Hickler, Y. Luo, Y. P. Wang, B. El-Masri, P. Thornton, Evaluation of 11 terrestrial carbon–nitrogen cycle models against observations from two temperate Free-Air CO_2 Enrichment studies. *New Phytol.* **202**, 803–822 (2014).

143. A. J. Bloom, F. S. Chapin III, H. A. Mooney, Resource limitation in plants-an economic analogy. *Annu. Rev. Ecol. Syst.* **16**, 363–392 (1985).

144. C. B. Field, F. S. Chapin III, P. A. Matson, H. A. Mooney, Responses of terrestrial ecosystems to the changing atmosphere: A resource-based approach. *Annu. Rev. Ecol. Syst.* **23**, 201–235 (1992).

145. O. Franklin, S. P. Harrison, R. Dewar, C. E. Farrior, Å. Brännström, U. Dieckmann, S. Pietsch, D. Falster, W. Cramer, M. Loreau, H. Wang, A. Mäkelä, K. T. Rebel, E. Meron, S. J. Schymanski, E. Rovenskaya, B. D. Stocker, S. Zaehle, S. Manzoni, M. van Oijen, I. J. Wright, P. Ciais, P. van Bodegom, J. Peñuelas, F. Hofhansl, C. Terrer, N. A. Soudzilovskaia, G. Midgley, I. C. Prentice, Organizing principles for vegetation dynamics. *Nat Plants* **6**, 444–453 (2020).

146. F. S. Chapin III, The mineral nutrition of wild plants. *Annu. Rev. Ecol. Syst.* **11**, 233–260 (1980).

147. B. A. Edgar, How flies get their size: Genetics meets physiology. *Nat. Rev. Genet.* **7**, 907–916 (2006).

148. A. Di Filippo, F. Biondi, M. Maugeri, B. Schirone, G. Piovesan, Bioclimate and growth history affect beech lifespan in the Italian Alps and Apennines. *Glob. Chang. Biol.* **18**, 960–972 (2012).

149. R. J. Brienen, L. Caldwell, L. Duchesne, S. Voelker, J. Barichivich, M. Baliva, G. Ceccantini, A. Di Filippo, S. Helama, G. M. Locosselli, Forest carbon sink neutralized by pervasive growth-lifespan trade-offs. *Nat. Commun.* **11**, 4241 (2020).

150. A. C. Bennett, N. G. McDowell, C. D. Allen, K. J. Anderson-Teixeira, Larger trees suffer most during drought in forests worldwide. *Nat Plants* **1**, 15139 (2015).

151. J. F. Needham, J. Chambers, R. Fisher, R. Knox, C. D. Koven, Forest responses to simulated elevated $_{CO(2)}$ under alternate hypotheses of size- and age-dependent mortality. *Glob. Chang. Biol.* **26**, 5734–5753 (2020).

152. E. M. Gora, A. Esquivel-Muelbert, Implications of size-dependent tree mortality for tropical forest carbon dynamics. *Nat Plants* **7**, 384–391 (2021).

153. J. M. Levine, J. HilleRisLambers, W. K. Petry, J. Usinowicz, T. W. Crowther, Demographic but not competitive time lags can transiently amplify climate-induced changes in vegetation carbon storage. *Glob. Chang. Biol.* **30**, e17432 (2024).

154. J. A. Hicke, M. J. Zeppel, Climate-driven tree mortality: Insights from the piñon pine die-off in the United States. *New Phytol.* **200**, 301–303 (2013).

155. T. A. Pugh, A. Arneth, M. Kautz, B. Poulter, B. Smith, Important role of forest disturbances in the global biomass turnover and carbon sinks. *Nat. Geosci.* **12**, 730–735 (2019).

156. W. R. Anderegg, A. T. Trugman, G. Badgley, C. M. Anderson, A. Bartuska, P. Ciais, D. Cullenward, C. B. Field, J. Freeman, S. J. Goetz, J. Hicke, D. Huntzinger, R. Jackson, J. Nickerson, S. Pacala, J. Randerson, Climate-driven risks to the climate mitigation potential of forests. *Science* **368**, eaaz7005 (2020).

157. D. van Wees, G. R. van Der Werf, J. T. Randerson, N. Andela, Y. Chen, D. C. Morton, The role of fire in global forest loss dynamics. *Glob. Chang. Biol.* **27**, 2377–2391 (2021).

158. C. D. Koven, Boreal carbon loss due to poleward shift in low-carbon ecosystems. *Nat. Geosci.* **6**, 452–456 (2013).

159. W. R. Anderegg, C. Wu, N. Acil, N. Carvalhais, T. A. Pugh, J. P. Sadler, R. Seidl, A climate risk analysis of Earth’s forests in the 21st century. *Science* **377**, 1099–1103 (2022).

160. V. K. Arora, A. Katavouta, R. G. Williams, C. D. Jones, V. Brovkin, P. Friedlingstein, J. Schwinger, L. Bopp, O. Boucher, P. Cadule, Carbon–concentration and carbon–climate feedbacks in CMIP6 models and their comparison to CMIP5 models. *Biogeosciences* **17**, 4173–4222 (2020).

161. N. G. McDowell, D. J. Beerling, D. D. Breshears, R. A. Fisher, K. F. Raffa, M. Stitt, The interdependence of mechanisms underlying climate-driven vegetation mortality. *Trends Ecol. Evol.* **26**, 523–532 (2011).

162. A. Trugman, M. Detto, M. Bartlett, D. Medvigy, W. Anderegg, C. Schwalm, B. Schaffer, S. W. Pacala, Tree carbon allocation explains forest drought-kill and recovery patterns. *Ecol. Lett.* **21**, 1552–1560 (2018).

163. A. T. Trugman, L. D. Anderegg, W. R. Anderegg, A. J. Das, N. L. Stephenson, Why is tree drought mortality so hard to predict? *Trends Ecol. Evol.* **36**, 520–532 (2021).

164. A. D. Friend, W. Lucht, T. T. Rademacher, R. Keribin, R. Betts, P. Cadule, P. Ciais, D. B. Clark, R. Dankers, P. D. Falloon, Carbon residence time dominates uncertainty in terrestrial vegetation responses to future climate and atmospheric CO_2. *Proc. Natl. Acad. Sci. U.S.A.* **111**, 3280–3285 (2014).

165. Z. Shi, S. D. Allison, Y. He, P. A. Levine, A. M. Hoyt, J. Beem-Miller, Q. Zhu, W. R. Wieder, S. Trumbore, J. T. Randerson, The age distribution of global soil carbon inferred from radiocarbon measurements. *Nat. Geosci.* **13**, 555–559 (2020).

166. Y. He, S. E. Trumbore, M. S. Torn, J. W. Harden, L. J. Vaughn, S. D. Allison, J. T. Randerson, Radiocarbon constraints imply reduced carbon uptake by soils during the 21st century. *Science* **353**, 1419–1424 (2016).

167. C. R. Lawrence, J. Beem-Miller, A. M. Hoyt, G. Monroe, C. A. Sierra, S. Stoner, K. Heckman, J. C. Blankinship, S. E. Crow, G. McNicol, An open-source database for the synthesis of soil radiocarbon data: International Soil Radiocarbon Database (ISRaD) version 1.0. *Earth Syst. Sci. Data* **12**, 61–76 (2020).

168. Y. Luo, A. Ahlström, S. D. Allison, N. H. Batjes, V. Brovkin, N. Carvalhais, A. Chappell, P. Ciais, E. A. Davidson, A. Finzi, Toward more realistic projections of soil carbon dynamics by Earth system models. *Global Biogeochem. Cycles* **30**, 40–56 (2016).

169. W. R. Wieder, G. B. Bonan, S. D. Allison, Global soil carbon projections are improved by modelling microbial processes. *Nat. Clim. Chang.* **3**, 909–912 (2013).

170. M. C. Hansen, P. Potapov, A. Tyukavina, Comment on "Tropical forests are a net carbon source based on aboveground measurements of gain and loss". *Science* **363**, eaar3629 (2019).

171. A. Neuenschwander, K. Pitts, The ATL08 land and vegetation product for the ICESat-2 Mission. *Remote Sens. Environ.* **221**, 247–259 (2019).

172. R. Dubayah, J. Armston, S. P. Healey, J. M. Bruening, P. L. Patterson, J. R. Kellner, L. Duncanson, S. Saarela, G. Ståhl, Z. Yang, GEDI launches a new era of biomass inference from space. *Environ. Res. Lett.* **17**, 095001 (2022).

173. K. Kellogg, P. Hoffman, S. Standley, S. Shaffer, P. Rosen, W. Edelstein, C. Dunn, C. Baker, P. Barela, Y. Shen, "NASA-ISRO synthetic aperture radar (NISAR) mission" in *2020 IEEE Aerospace Conference* (IEEE, 2020), pp. 1–21.

174. S. Quegan, T. Le Toan, J. Chave, J. Dall, J.-F. Exbrayat, D. H. T. Minh, M. Lomas, M. M. D'alessandro, P. Paillou, K. Papathanassiou, The European Space Agency BIOMASS mission: Measuring forest above-ground biomass from space. *Remote Sens. Environ.* **227**, 44–60 (2019).

175. K. Novick, T. Keenan, W. Anderegg, C. Normile, B. Runkle, E. Oldfield, G. Shrestha, D. Baldocchi, M. Evans, J. Randerson, J. Sanderman, M. Torn, A. Trugman, C. Williams, We need a solid scientific basis for nature-based climate solutions in the United States. *Proc. Natl. Acad. Sci. U.S.A.* **121**, e2318505121 (2024).

176. G. Grassi, G. Conchedda, S. Federici, R. A. Viñas, A. Korosuo, J. Melo, S. Rossi, M. Sandker, Z. Somogyi, M. Vizzarri, F. N. Tubiello, Carbon fluxes from land 2000–2020: Bringing clarity to countries' reporting. *Earth Syst. Sci. Data* **14**, 4643–4666 (2022).

177. N. R. C. U. S. B. o. A. S. a. C. National Research Council (U.S.), Committee on Methods for Estimating Greenhouse Gas Emissions, National Research Council (U.S.). Division on Earth and Life Studies, "Verifying greenhouse gas emissions: Methods to support international climate agreements" (National Academies Press, 2010).

178. D. J. Jacob, A. J. Turner, J. D. Maasakkers, J. Sheng, K. Sun, X. Liu, K. Chance, I. Aben, J. McKeever, C. Frankenberg, Satellite observations of atmospheric methane and their value for quantifying methane emissions. *Atmos. Chem. Phys.* **16**, 14371–14396 (2016).

179. D. H. Cusworth, D. J. Jacob, D. J. Varon, C. Chan Miller, X. Liu, K. Chance, A. K. Thorpe, R. M. Duren, C. E. Miller, D. R. Thompson, Potential of next-generation imaging spectrometers to detect and quantify methane point sources from space. *Atmospheric Meas. Tech.* **12**, 5655–5668 (2019).

180. X. Ye, T. Lauvaux, E. A. Kort, T. Oda, S. Feng, J. C. Lin, E. G. Yang, D. Wu, Constraining fossil fuel CO_2 emissions from urban area using OCO-2 observations of total column CO_2. *J. Geophys. Res. Atmos.* **125**, e2019JD030528 (2020).

181. R. Nassar, J.-P. Mastrogiacomo, W. Bateman-Hemphill, C. McCracken, C. G. MacDonald, T. Hill, C. W. O'Dell, M. Kiel, D. Crisp, Advances in quantifying power plant CO_2 emissions with OCO-2. *Remote Sens. Environ.* **264**, 112579 (2021).

182. C. Tucker, M. Brandt, P. Hiernaux, A. Kariryaa, K. Rasmussen, J. Small, C. Igel, F. Reiner, K. Melocik, J. Meyer, S. Sinno, E. Romero, E. Glennie, Y. Fitts, A. Morin, J. Pinzon, D. McClain, P. Morin, C. Porter, S. Loeffler, L. Kergoat, B. A. Issoufou, P. Savadogo, J. P. Wigneron, B. Poulter, P. Ciais, R. Kaufmann, R. Myneni, S. Saatchi, R. Fensholt, Sub-continental-scale carbon stocks of individual trees in African drylands. *Nature* **615**, 80–86 (2023).

183. G. Grassi, J. House, W. A. Kurz, A. Cescatti, R. A. Houghton, G. P. Peters, M. J. Sanz, R. A. Viñas, R. Alkama, A. Arneth, Reconciling global-model estimates and country reporting of anthropogenic forest CO_2 sinks. *Nat. Clim. Chang.* **8**, 914–920 (2018).

184. T. DeVries, M. Holzer, Radiocarbon and helium isotope constraints on deep ocean ventilation and mantle-^{3}He sources. *J. Geophys. Res. Oceans* **124**, 3036–3057 (2019).

185. P. Suntharalingam, J. T. Randerson, N. Krakauer, J. A. Logan, D. J. Jacob, Influence of reduced carbon emissions and oxidation on the distribution of atmospheric CO_2: Implications for inversion analyses. *Global Biogeochem. Cycles* **19**, doi.org/10.1029/2005GB002466 (2005).

186. L. Resplandy, R. Keeling, C. Rödenbeck, B. Stephens, S. Khatiwala, K. Rodgers, M. Long, L. Bopp, P. Tans, Revision of global carbon fluxes based on a reassessment of oceanic and riverine carbon transport. *Nat. Geosci.* **11**, 504–509 (2018).

187. A. S. Denning, I. Y. Fung, D. Randall, Latitudinal gradient of atmospheric CO_2 due to seasonal exchange with land biota. *Nature* **376**, 240–243 (1995).

188. L. Resplandy, R. F. Keeling, Y. Eddebbar, M. Brooks, R. Wang, L. Bopp, M. C. Long, J. P. Dunne, W. Koeve, A. Oschlies, Quantification of ocean heat uptake from changes in atmospheric O_2 and CO_2 composition. *Sci. Rep.* **9**, 20244 (2019).

189. J. T. Randerson, C. Masiello, C. Still, T. Rahn, H. Poorter, C. Field, Is carbon within the global terrestrial biosphere becoming more oxidized? Implications for trends in atmospheric O_2. *Glob. Chang. Biol.* **12**, 260–271 (2006).

190. T. Ziehn, M. A. Chamberlain, R. M. Law, A. Lenton, R. W. Bodman, M. Dix, L. Stevens, Y.-P. Wang, J. Srbinovsky, The Australian Earth System Model: ACCESS-ESM1. 5. *J. South. Hemisph. Earth Syst. Sci.* **70**, 193–214 (2020).

191. T. Wu, W. Li, J. Ji, X. Xin, L. Li, Z. Wang, Y. Zhang, J. Li, F. Zhang, M. Wei, Global carbon budgets simulated by the Beijing Climate Center Climate System Model for the last century. *J. Geophys. Res. Atmos.* **118**, 4326–4347 (2013).

192. N. C. Swart, J. N. Cole, V. V. Kharin, M. Lazare, J. F. Scinocca, N. P. Gillett, J. Anstey, V. Arora, J. R. Christian, S. Hanna, The Canadian Earth System Model version 5 (CanESM5. 0.3). *Geosci. Model Dev.* **12**, 4823–4873 (2019).

193. M. Sigmond, J. Anstey, V. Arora, R. Digby, N. Gillett, V. Kharin, W. Merryfield, C. Reader, J. Scinocca, N. Swart, Improvements in the Canadian Earth System Model (CanESM) through systematic model analysis: CanESM5. 0 and CanESM5. 1. *Geosci. Model Dev.* **16**, 6553–6591 (2023).

194. G. Danabasoglu, J. F. Lamarque, J. Bacmeister, D. Bailey, A. DuVivier, J. Edwards, L. Emmons, J. Fasullo, R. Garcia, A. Gettelman, The Community Earth System Model version 2 (CESM2). *J. Adv. Model. Earth Syst.* **12**, e2019MS001916 (2020).

195. A. Cherchi, P. G. Fogli, T. Lovato, D. Peano, D. Iovino, S. Gualdi, S. Masina, E. Scoccimarro, S. Materia, A. Bellucci, Global mean climate and main patterns of variability in the CMCC-CM2 coupled model. *J. Adv. Model. Earth Syst.* **11**, 185–209 (2019).

196. R. Séférian, P. Nabat, M. Michou, D. Saint-Martin, A. Voldoire, J. Colin, B. Decharme, C. Delire, S. Berthet, M. Chevallier, Evaluation of CNRM Earth System Model, CNRM-ESM2-1: Role of Earth system processes in present-day and future climate. *J. Adv. Model. Earth Syst.* **11**, 4182–4227 (2019).

197. R. Döscher, M. Acosta, A. Alessandri, P. Anthoni, T. Arsouze, T. Bergman, R. Bernardello, S. Boussetta, L. P. Caron, G. Carver, M. Castrillo, F. Catalano, I. Cvijanovic, P. Davini, E. Dekker, F. J. Doblas-Reyes, D. Docquier, P. Echevarria, U. Fladrich, R. Fuentes-Franco, M. Gröger, J. v. Hardenberg, J. Hieronymus, M. P. Karami, J. P. Keskinen, T. Koenigk, R. Makkonen, F. Massonnet, M. Ménégoz, P. A. Miller, E. Moreno-Chamarro, L. Nieradzik, T. van Noije, P. Nolan, D. O'Donnell, P. Ollinaho, G. van den Oord, P. Ortega, O. T. Prims, A. Ramos, T. Reerink, C. Rousset, Y. Ruprich-Robert, P. Le Sager, T. Schmith, R. Schrödner, F. Serva, V. Sicardi, M. S. Madsen, B. Smith, T. Tian, E. Tourigny, P. Uotila, M. Vancoppenolle, S. Wang, D. Wårlind, U. Willén, K. Wyser, S. Yang, X. Yepes-Arbós, Q. Zhang, The EC-Earth3 Earth system model for the Coupled Model Intercomparison Project 6. *Geosci. Model Dev.* **15**, 2973–3020 (2022).

198. O. Boucher, J. Servonnat, A. L. Albright, O. Aumont, Y. Balkanski, V. Bastrikov, S. Bekki, R. Bonnet, S. Bony, L. Bopp, Presentation and evaluation of the IPSL-CM6A-LR climate model. *J. Adv. Model. Earth Syst.* **12**, e2019MS002010 (2020).

199. T. Hajima, M. Watanabe, A. Yamamoto, H. Tatebe, M. A. Noguchi, M. Abe, R. Ohgaito, A. Ito, D. Yamazaki, H. Okajima, Development of the MIROC-ES2L earth system model and the evaluation of biogeochemical processes and feedbacks. *Geosci. Model Dev.* **13**, 2197–2244 (2020).

200. K.-H. Wieners, M. Giorgetta, J. Jungclaus, C. Reick, M. Esch, M. Bittner, S. Legutke, M. Schupfner, F. Wachsmann, V. Gayler, H. Haak, P. de Vrese, T. Raddatz, T. Mauritsen, J.-S. von Storch, J. Behrens, V. Brovkin, M. Claussen, T. Crueger, I. Fast, S. Fiedler, S. Hagemann, C.

Hohenegger, T. Jahns, S. Kloster, S. Kinne, G. Lasslop, L. Kornblueh, J. Marotzke, D. Matei, K. Meraner, U. Mikolajewicz, K. Modali, W. Müller, J. Nabel, D. Notz, K. Peters-von Gehlen, R. Pincus, H. Pohlmann, J. Pongratz, S. Rast, H. Schmidt, R. Schnur, U. Schulzweida, K. Six, B. Stevens, A. Voigt, E. Roeckner, MPI-M MPI-ESM1.2-LR model output prepared for CMIP6 CMIP historical. https://doi.org/10.22033/ESGF/CMIP22036.26595 (2019).

201. T. Mauritsen, E. Roeckner, Tuning the MPI-ESM1. 2 global climate model to improve the match with instrumental record warming by lowering its climate sensitivity. *J. Adv. Model Earth Syst.* **12**, e2019MS002037 (2020).

202. Ø. Seland, M. Bentsen, D. Olivié, T. Toniazzo, A. Gjermundsen, L. S. Graff, J. B. Debernard, A. K. Gupta, Y.-C. He, A. Kirkevåg, Overview of the Norwegian Earth System Model (NorESM2) and key climate response of CMIP6 DECK, historical, and scenario simulations. *Geosci. Model Dev.* **13**, 6165–6200 (2020).

203. A. A. Sellar, C. G. Jones, J. P. Mulcahy, Y. Tang, A. Yool, A. Wiltshire, F. M. O'connor, M. Stringer, R. Hill, J. Palmieri, UKESM1: Description and evaluation of the UK Earth System Model. *J. Adv. Model Earth Syst.* **11**, 4513–4558 (2019).
